# OTUB1/NDUFS2 axis promotes pancreatic tumorigenesis through protecting against mitochondrial cell death

**DOI:** 10.1038/s41420-024-01948-x

**Published:** 2024-04-23

**Authors:** Xiao-Dong Huang, Li Du, Xiao-Chen Cheng, Yu-Xin Lu, Qiao-Wei Liu, Yi-Wu Wang, Ya-Jin Liao, Dong-Dong Lin, Feng-Jun Xiao

**Affiliations:** 1 Department of General Surgery, Xuanwu Hospital Capital Medical University, Beijing, 100053 PR China; 2 Department of Experimental Hematology and Biochemistry, Beijing Institute of Radiation Medicine, Beijing, 100850 PR China; 3 Department of Oncology, Fifth Medical Center, Chinese PLA General Hospital, Beijing, 100039 PR China; 4 Department of Disease Control and Prevention, Chinese PLA The 96601 Military Hospital, Huangshan, 242700 Anhui PR China; 5 Department of Neurology, The Second Affiliated Hospital, Hengyang Medical School, University of South China, Hengyang, 42100 Hunan PR China

**Keywords:** Prognostic markers, Tumour biomarkers

## Abstract

Pancreatic cancer is one of the most fatal cancers in the world. A growing number of studies have begun to demonstrate that mitochondria play a key role in tumorigenesis. Our previous study reveals that *NDUFS2* (NADH: ubiquinone oxidoreductase core subunit S2), a core subunit of the mitochondrial respiratory chain complex I, is upregulated in Pancreatic adenocarcinoma (PAAD). However, its role in the development of PAAD remains unknown. Here, we showed that *NDUFS2* played a critical role in the survival, proliferation and migration of pancreatic cancer cells by inhibiting mitochondrial cell death. Additionally, protein mass spectrometry indicated that the *NDUFS2* was interacted with a deubiquitinase, *OTUB1*. Overexpression of *OTUB1* increased *NDUFS2* expression at the protein level, while knockdown of *OTUB1* restored the effects in vitro. Accordingly, overexpression and knockdown of *OTUB1* phenocopied those of *NDUFS2* in pancreatic cancer cells, respectively. Mechanically, *NDUFS2* was deubiquitinated by OTUB1 via K48-linked polyubiquitin chains, resulted in an elevated protein stability of NDUFS2. Moreover, the growth of *OTUB1*-overexpressed pancreatic cancer xenograft tumor was promoted in vivo, while the *OTUB1*-silenced pancreatic cancer xenograft tumor was inhibited in vivo. In conclusion, we revealed that *OTUB1* increased the stability of *NDUFS2* in PAAD by deubiquitylation and this axis plays a pivotal role in pancreatic cancer tumorigenesis and development.

## Introduction

PAAD is the fourth leading cause of all cancer-related deaths, with an overall 5-year survival rate less than 10%, and it is expected to occupy the second place in 2030 [1, 2]. The poor prognosis of PAAD is majorly attributed to the late diagnosis, frequent metastases, and limited treatment options [3]. Up to date, early diagnosis and prompt surgical intervention are the only effective means of improving the prognosis of those patients [4]. Unfortunately, even with a successful first-stage surgical resection, the prognosis is still poor, with a no more than 42% 2-year survival rate due to the high recurrence rate [5]. However, the underlying mechanism is poorly understood.

Mitochondria play a crucial role in cellular bioenergetics and apoptosis, and thus are important to support cell function and in determination of cell death pathways [6]. The alterations in mitochondrial function and dynamics have been connected with metabolic diseases, neurological disorders and cancer progression as well [7]. *NDUFS2*, encoded by nuclear DNA, is localized in mitochondria and is one of the core subunits of the mitochondrial respiratory chain complex I. It has been shown that interfering with the intracellular NDUFS2 inhibited the activity of Complex I, reduced the intracellular ATP production, and thereby suppressed tumor growth and metastasis [8]. Elevated expression of *NDUFS2* is associated with overall survival of several kinds of cancers, including lung, breast, and ovarian cancers [9]. It has been reported that the *NDUFS2* is a prognostic biomarker of lung cancer and it has the potential to be widely used in future clinical settings [10]. Our previous study has demonstrated that *NDUFS2* levels were elevated in pancreatic cancer tissues compared to adjacent normal tissues [11]. However, the role of *NDUFS2* in pancreatic cancers remains uncovered.

The ubiquitin system is an enzymatic cascade that conjugant amino ubiquitin or polyubiquitin chains to target proteins, thereby promoting degradation or changing their activities [12]. Deubiquitinases (DUBs), a large class of proteases that cleaves mono- or polyubiquitin from target proteins, are considered to be a potential therapeutic target for various diseases [13, 14]. *OTUB1*, one of the protein deubiquitinases, is widely involved in various physiological and pathological processes and abundant expressed in many human tumors such as colorectal, lung, liver, ovarian and esophageal cancers [15–19], which is also strongly associated with the prognosis of those diseases. A recent report showed that *OTUB1* could accelerate metastasis of pancreatic cancer by inhibiting *FOXM1* degradation [20]. But its role in pancreatic cancers has not yet been fully demonstrated.

Considering the importance of *OTUB1* and *NDUFS2* in mitochondrial metabolism and tumorigenesis, we hypothesized that they may be involved in mitochondrial functions and tumorigenesis of pancreatic cancer. Thus, pancreatic cell lines and clinical surgical specimens were collected to verify our hypothesis. The primary aim of this study was to investigate whether *OTUB1* or *NDUFS2* or both of them can be used as new prognostic biomarkers and/or therapeutic targets for pancreatic cancer and shed new lights into exploring therapeutic methods for pancreatic cancer.

## Results

### NDUFS2 promotes pancreatic cancer cell growth, proliferation and migration

**Fig. 1:**
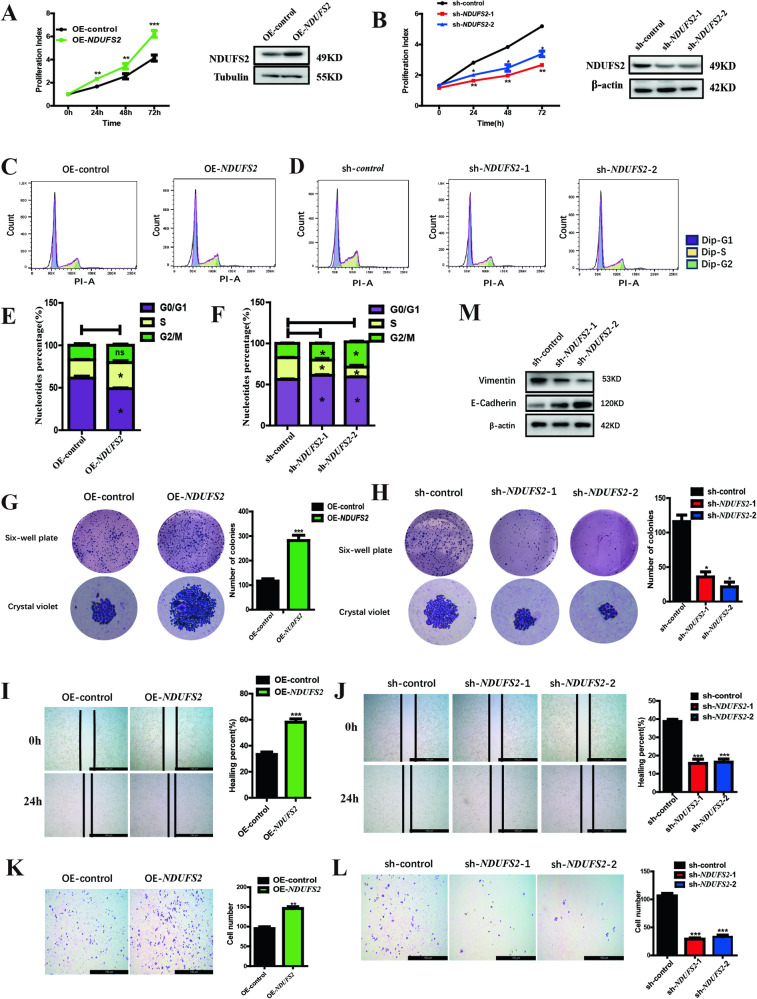
*NDUFS2* promotes the proliferation and migration of pancreatic cancer cell lines. **A** CCK-8 and western blotting assay shows the proliferation index and transduction rate in Panc05.04 cells transfected with OE-*NDUFS2* and control plasmids. **B** CCK-8 and western blotting assay shows the proliferation index and transduction rate in Panc05.04 cells transfected with sh-*NDUFS2* and control plasmids. **C** Cell-cycle assay shows the percentage of cell-cycle distribution of Panc05.04 cells transfected with OE-*NDUFS2* and control plasmids. **D** Cell-cycle assay shows the percentage of cell-cycle distribution of Panc05.04 cells transfected with sh-*NDUFS2* and control plasmids. **E** The statistical result of cell-cycle assay in Panc05.04 cells transfected with OE-*NDUFS2* and control plasmids. **F** The statistical result of cell-cycle assay in Panc05.04 cells transfected with sh-*NDUFS2* and control plasmids. **G** Cell colony formation assay shows the colony size in Panc05.04 cells transfected with OE-*NDUFS2* and control plasmids. **H** Cell colony formation assay shows the colony size in Panc05.04 cells transfected with sh-*NDUFS2* and control plasmids. **I** Wound healing assay shows the wound healing rate in Panc05.04 cells transfected with OE-*NDUFS2* and control plasmids. **J** Wound healing assay shows the wound healing rate in Panc05.04 cells transfected with sh-*NDUFS2* and control plasmids. **K** Transwell migration assay shows the number of transitional cells that migrated to the bottom of the chamber in Panc05.04 cells transfected with OE-*NDUFS2* and control plasmids. **L** Transwell migration assay shows the number of transitional cells that migrated to the bottom of the chamber in Panc05.04 cells transfected with sh-*NDUFS2* and control plasmids. **M** Western blotting shows the expression of E-cadherin and Vimentin in Panc05.04 cells transfected with sh-*NDUFS2* and control plasmids.

In our previous study, we identified *NDUFS2* as a downstream factor of *PTPMT1* in pancreatic cancer [11], but the precise role has not yet been validated. To determine the biological function of *NDUFS2* in pancreatic cancer, overexpression and knockdown assays were performed in pancreatic cancer cell lines, Panc05.04 and ASPC-1. The results shows that overexpression of *NDUFS2* significantly promoted the cell proliferation, whereas knockdown of it apparently inhibited the proliferation, revealed by CCK-8 assay (Fig. 1A, B left panel). The knockdown and overexpression efficiencies were verified by Western blot (Fig. 1A, B right panel). Consistently, flow cytometric analysis showed that overexpression of *NDUFS2* notably promoted the cell-cycle progression exhibited by elevated S-phase percentage (Fig. 1C, E), and it was impeded at G0/G1 phase in *NDUFS2*-silenced cells (Fig. 1D, F). Additionally, colony formation capability of *NDUFS2*-overexpressed cells was significantly enhanced in contrast to the control (Fig. 1G), and it was dampened when *NDUFS2* was knocked down compared to the control (Fig. 1H). Furthermore, wound healing and transwell migration assays showed that the mobility of *NDUFS2*-overexpressed cells was markedly increased, whereas that of *NDUFS2*-silenced cells was weakened (Fig. 1I–L). Accordingly, the expression of E-cadherin was upregulated and Vimentin was downregulated compared to the control in *NDUFS2*-silenced cells (Fig. 1M). Those results indicated a critical role of *NDUFS2* in pancreatic cancer cell growth, cell-cycle and migration.

### *NDUFS2* is an essential factor in mitochondrial membrane dynamics and ATP production in pancreatic cancer cells

**Fig. 2:**
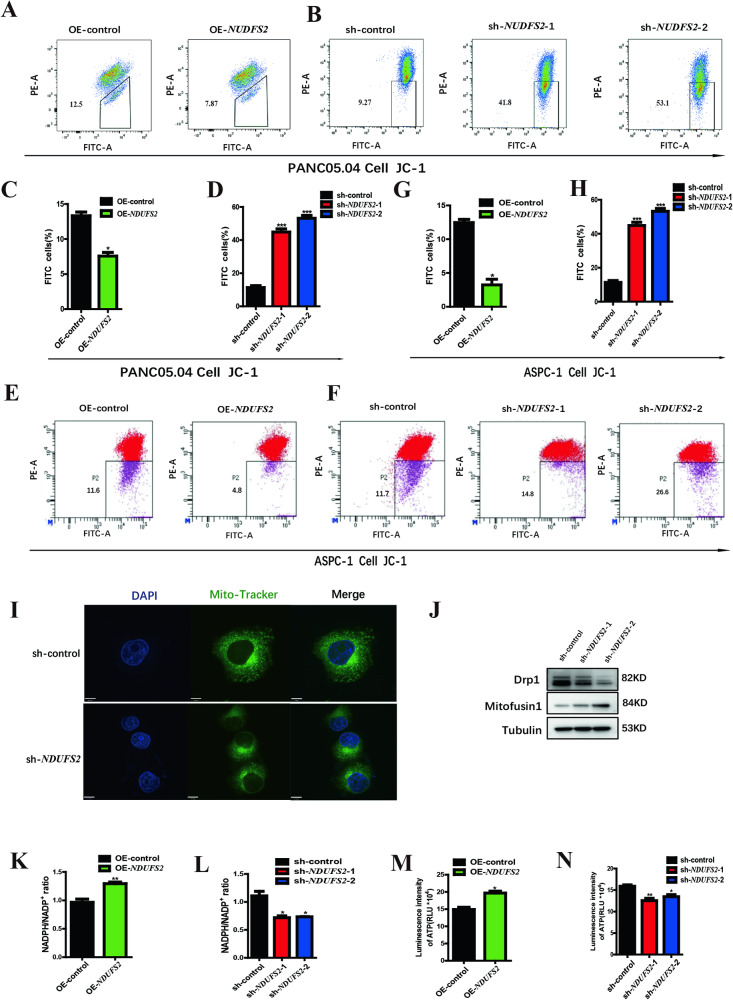
*NDUFS2* suppresses mitochondrial death and promotes ATP synthesis in pancreatic cancer cell lines. **A** FACS analysis shows the MMP changes in Panc05.04 cells transfected with OE-*NDUFS2* and control plasmids. **B** FACS analysis shows the MMP changes in Panc05.04 cells transfected with sh-*NDUFS2* and control plasmids. **C** The statistical result of MMP changes in Panc05.04 cells transfected with OE-*NDUFS2* and control plasmids. **D** The statistical result of MMP changes in Panc05.04 cells transfected with sh-*NDUFS2* and control plasmids. **E** FACS analysis shows the MMP changes in ASPC-1 cells transfected with OE-*NDUFS2* and control plasmids. **F** FACS analysis shows the MMP changes in ASPC-1 cells transfected with sh-*NDUFS2* and control plasmids. **G** The statistical result of MMP changes in ASPC-1 cells transfected with OE-*NDUFS2* and control plasmids. **H** The statistical result of MMP changes in ASPC-1 cells transfected with sh-*NDUFS2* and control plasmids. **I** Mito-tracker staining shows the changes in mitochondrial morphology in Panc05.04 cell transfected with sh-*NDUFS2* and control plasmids. **J** Western blotting shows the expression of Drp1 and Mfn2 in Panc05.04 cells transfected with sh-*NDUFS2* and control plasmids. **K** NADP^+^/NADPH ratios in the culture media were measured in Panc05.04 cells transfected with OE-*NDUFS2* and control plasmids. **L** NADP^+^/NADPH ratios in the culture media were measured in Panc05.04 cells transfected with sh-*NDUFS2* and control plasmids. **M** The ATP concentration in the culture media was measured in Panc05.04 cell transfected with OE-*NDUFS2* and control plasmids. **N** The ATP concentration in the culture media was measured in Panc05.04 cell transfected with sh-*NDUFS2* and control plasmids.

Given that *NDUFS2* is a core subunit of the mitochondrial respiratory chain complex I, we inferred that it may regulate such cellular processes through modulating mitochondria. Therefore, mitochondrial membrane potential (MMP) was measured by JC-1 in *NDUFS2*-overexpressed and -silenced cells, respectively. Overexpression of *NDUFS2* significantly increased the MMP in Panc05.04 cells, and thereby inhibited mitochondrial cell death (Fig. 2A, C). Accordingly, knockdown of *NDUFS2* markedly decreased the MMP, accompanied by aggravated mitochondrial cell death (Fig. 2B, D). Similar results were observed in another pancreatic cancer cell line, Aspc-1 (Fig. 2E–H). In addition, confocal analysis revealed that the mitochondria, stained by Mito-Tracker, in the cytoplasm of *NDUFS2*-silenced cells were considerably shorter and fewer than those in the control cells (Fig. 2I). Those results suggested that *NDUFS2* has a pivotal role in mitochondrial dynamics and cell fate determination in pancreatic cancer cells. Mitochondrial dynamics encompass processes of fusion and fission, with *Drp1* participating in fission, while mitofusins(*Mfn1*) is associated with fusion [21].We also examined the expression of *Drp1* and *Mfn1* after knockdown of *NDUFS2*.The result showed that the expression of Drp1 was downregulated while *Mfn1* was upregulated, which means intracellular mitochondrial division decreased and mitochondrial fusion increased (Fig. 2J). This causes a decrease in the number of mitochondria in the cells.

To further validate the effects of *NDUFS2* on cellular redox function and energy production, the NADPH/NADP^+^ ratio and ATP concentration in the culture media were measured. Overexpression of *NDUFS2* increased the NADPH/NADP^+^ ratio and ATP concentration in Panc05.04 cells in contrast to the control (Fig. 2K, M), and knockdown of it decreased the NADPH/NADP^+^ ratio and ATP concentration in accordance (Fig. 2L, N), suggesting that *NDUFS2* plays a key role in mitochondrial functions and redox system. Collectively, those data suggested that *NDUFS2* plays an indispensable role in mitochondrial function and homeostasis in pancreatic cancer cells, which determined the cell fate.

### *OTUB1* interacts with *NDUFS2* in pancreatic cancer

**Fig. 3:**
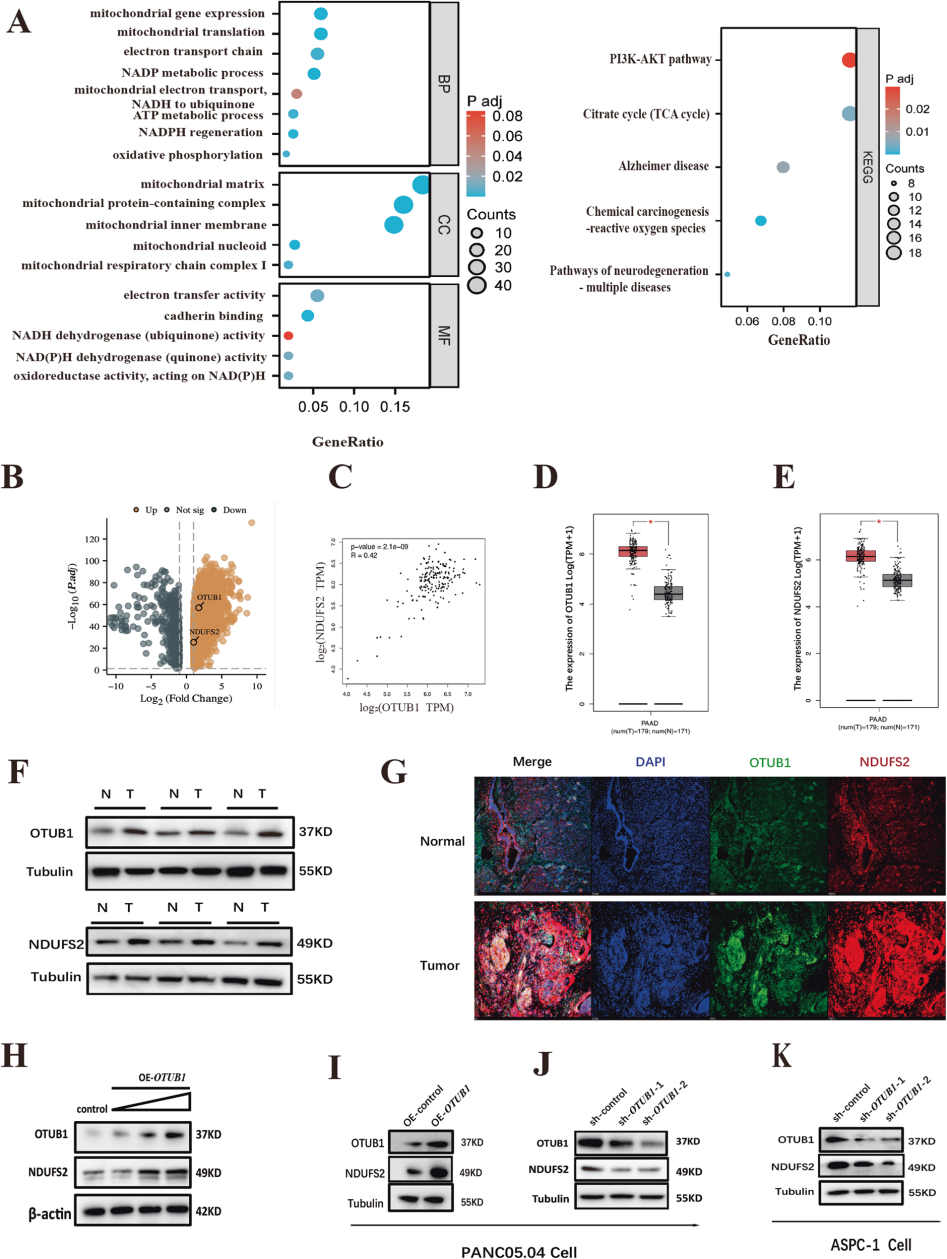
*NDUFS2* interact with *OTUB1* in the mitochondrial death of pancreatic cancer. **A** GO and KEGG pathway analysis after overexpression of *NDUFS2*. **B** The volcanic map depicting the dysfunctional genes in pancreatic cancer and the location of *OTUB1* and *NDUFS2*. **C** The correlation between *OTUB1* and *NDUFS2* was analyzed according to the TCGA database online (*R* = 0.42, *P* < 0.01). **D** The transcriptional expression of *OTUB1* in pancreatic cancer. **E** The transcriptional expression of *NDUFS2* in pancreatic cancer. **F** The expression of *OTUB1* and *NDUFS2* in pancreatic cancer and normal control. **G** Confocal analysis shows the expression of *OTUB1* and *NDUFS2* in in pancreatic cancer and normal control (Scale bar: 10 μm; magnification: ×100). **H** Western blotting detecting the expression of *NDUFS2* when upregulated *OTUB1* expression in Panc05.04 cells. **I** Western blotting detecting the expression of *NDUFS2* when transfected with OE-*NDUFS2* and control plasmids in Panc05.04 cells. **J** Western blotting detecting the expression of *NDUFS2* when transfected with sh-*OTUB1* and control plasmids in Panc05.04 cells. **K** Western blotting detecting the expression of *NDUFS2* when transfected with sh-*OTUB1* and control plasmids in ASPC-1 cells.

In order to find out the possible protein that interact with *NDUFS2* in pancreatic cancer, co-immuno-precipitation (co-IP) was carried out to capture *NDUFS2* interactors. We identified 17,726 potential interactors using protein mass spectrometry. Next, we subjected these candidate interactors to GO and KEGG pathway analysis and identified the top 10 terms regulated by *NDUFS2* as shown in Fig. 3A and Table 1. These results indicate that potential *NDUFS2*-interacting proteins are tightly associated with mitochondrial function and PI3K-AKT pathway. Among all the unique peptides, 10 target genes were selected for further experimental validation. Then, we filtered all the upregulated and downregulated genes in pancreatic cancer in the online TCGA database, and identified that *OTUB1* and *NDUFS2* were both upregulated in pancreatic cancer among all the upregulated genes, deciphered by the volcano map (Fig. 3B). Meanwhile, we also found that the level of *NDUFS2* was positively correlated with *OTUB1* via GEPIA online database (*R* = 0.42; *P* < 0.01; Fig. 3C). Statistical analysis indicated that the expression of *OTUB1* and *NDUFS2* in pancreatic cancers was significantly higher than those in normal pancreatic tissues at transcriptional level (Fig. 3D, E). To further confirm these results, pancreatic cancer and adjacent tissue specimens were collected during clinical surgery, western blot assay was performed to measure the expression of OTUB1 and NDUFS2. The result showed that OTUB1, as well as NDUFS2, were abundantly expressed in pancreatic cancers in contrast to the adjacent tissues (Fig. 3F). Confocal assay showed that *OTUB1* and *NDUFS2* were co-localized and upregulated in pancreatic cancer tissues compared to the adjacent normal tissues (Fig. 3G). Further experiments indicated that overexpression of *OTUB1* in pancreatic cancer cells increased the expression of NDUFS2 (Fig. 3H, I), and knockdown of *OTUB1* decreased the expression of *NDUFS2* in vitro (Fig. 3J, K). These results suggested that *OTUB1* could be a co-regulator of *NDUFS2* in pancreatic cancer progression.

**Table 1:** Protein mass spectrometry analysis the possible interaction proteins of NDUFS2 after Co-IP.

Gene	Description	Coverage	Peptides	PSMs	Unique Peptides
OTUB1	OTU deubiquitinase, ubiquitin aldehyde binding 1 [*Homo sapiens*]	16.33121	39	251	40
NDUFS3	NADH dehydrogenase [ubiquinone] iron-sulfur protein 3, mitochondrial precursor [*Homo sapiens*]	52.51572	40	122	28
TFB1M	dimethyladenosine transferase 1, mitochondrial [*Homo sapiens*]	27.67978	45	135	26
AKR1A1	alcohol dehydrogenase [NADP(+)] [*Homo sapiens*]	35.47672	60	95	30
TIMM13	mitochondrial import inner membrane translocase subunit Tim13 [*Homo sapiens*]	25.42857	33	101	11
ACADL	long-chain specific acyl-CoA dehydrogenase, mitochondrial precursor [*Homo sapien*s]	19.07731	18	76	7
PSMA7	proteasome subunit alpha type-7 [*Homo sapiens*]	23.36245	35	71	32
SLC25A11	mitochondrial 2-oxoglutarate/malate carrier protein isoform 1 [*Homo sapiens*]	17.10526	39	59	35
HMGCS2	hydroxymethylglutaryl-CoA synthase, mitochondrial isoform 1 precursor [*Homo sapiens*]	18.08511	35	112	5
MRPL39	39S ribosomal protein L39, mitochondrial isoform b [*Homo sapiens*]	20.16129	34	135	3

### *OTUB1* mimicked the role of *NDUFS2* in modulating cell growth, proliferation and migration in pancreatic cancer cells

**Fig. 4:**
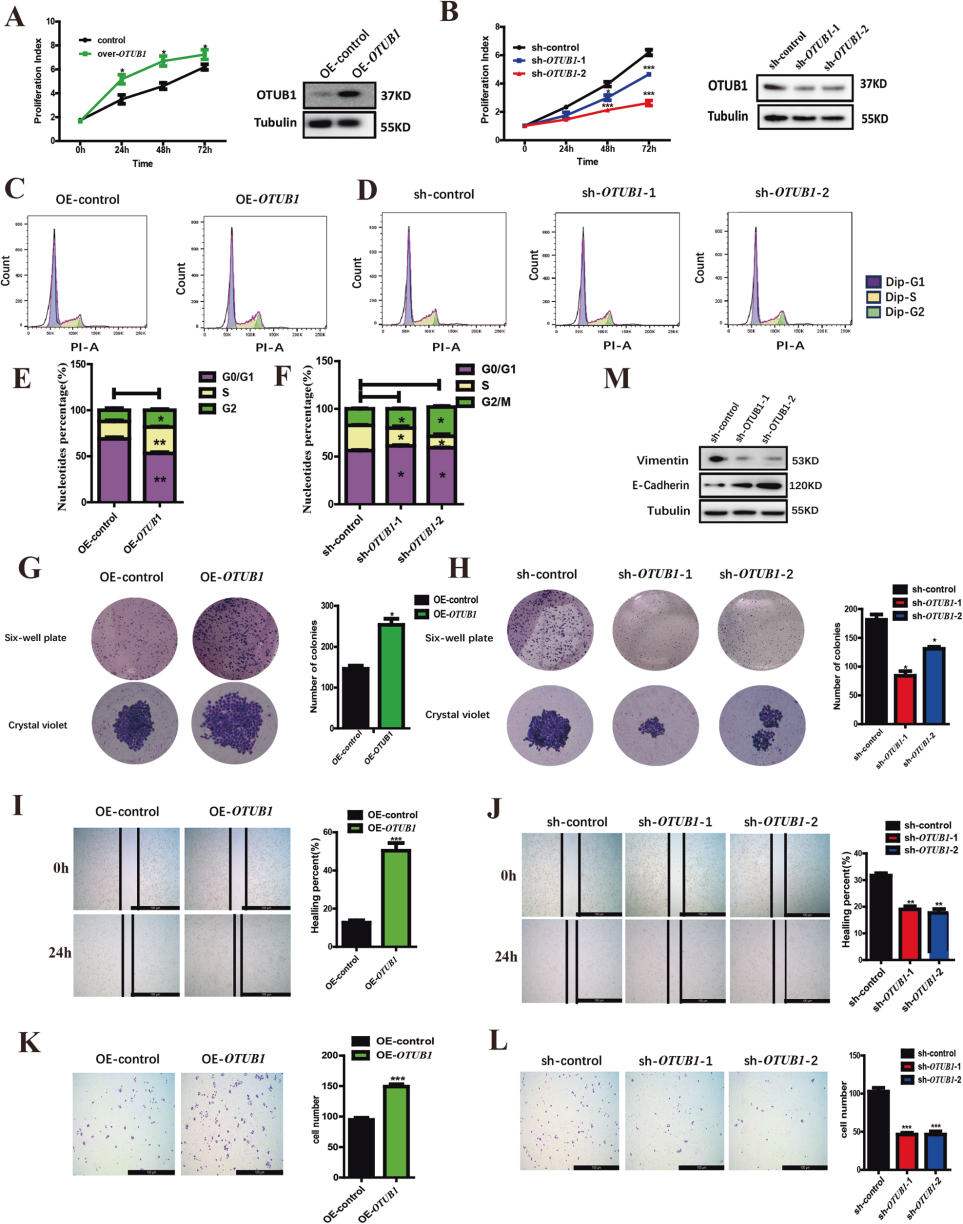
*OTUB1* promotes the proliferation and migration of pancreatic cancer cell lines. **A** CCK-8 and western blotting assay shows the proliferation index and transduction rate in Panc05.04 cells transfected with OE- *OTUB1* and control plasmids. **B** CCK-8 and western blotting assay shows the proliferation index and transduction rate in Panc05.04 cells transfected with sh-*OTUB1* and control plasmids. **C** Cell-cycle assay shows the percentage of cell-cycle distribution of Panc05.04 cells transfected with OE-*OTUB1* and control plasmids. **D** Cell-cycle assay shows the percentage of cell-cycle distribution of Panc05.04 cells transfected with sh-*OTUB1* and control plasmids. **E** The statistical result of cell-cycle assay in Panc05.04 cells transfected with OE-*OTUB1* and control plasmids. **F** The statistical result of cell-cycle assay in Panc05.04 cells transfected with sh-*OTUB1* and control plasmids. **G** Cell colony formation assay shows the colony size in Panc05.04 cells transfected with OE-*OTUB1* and control plasmids. **H** Cell colony formation assay shows the colony size in Panc05.04 cells transfected with sh-*OTUB1* and control plasmids. **I** Wound healing assay shows the wound healing rate in Panc05.04 cells transfected with OE-*OTUB1* and control plasmids. **J** Wound healing assay shows the wound healing rate in Panc05.04 cells transfected with sh-*OTUB1* and control plasmids. **K** Transwell migration assay shows the number of transitional cells that migrated to the bottom of the chamber in Panc05.04 cells transfected with OE-*OTUB1* and control plasmids. **L** Transwell migration assay shows the number of transitional cells that migrated to the bottom of the chamber in Panc05.04 cells transfected with sh-*OTUB1* and control plasmids. **K** Western blotting shows the expression of E-cadherin and Vimentin in Panc05.04 cells transfected with sh-*OTUB1* and control plasmids.

According the result of the protein mass spectrometry, we conjectured that the effects of *OTUB1* on cell phenotype may be similar with *NDUFS2*. Overexpression and knockdown assays were performed in Panc05.04 cells to verify our assumption. The results showed that overexpression of *OTUB1* significantly promoted cell proliferation, whereas knockdown of it apparently inhibited the proliferation, revealed by CCK-8 assay (Fig. 4A, B left panel). The knockdown and overexpression efficiencies were verified by Western blot (Fig. 4A, B right panel). Consistently, flow cytometric analysis showed that overexpression of OTUB1 notably promoted the cell-cycle progression exhibited by elevated S-phase percentage (Fig. 4C, E), and it was impeded at G0/G1 phase in *OTUB1*-scilenced cells (Fig. 4D, F). Additionally, colony formation capability of *OTUB1*-overexpressed cells was enhanced in contrast to the control (Fig. 4G), and it was dampened when *OTUB1* was knocked down compared to the control (Fig. 4H). Furthermore, wound healing and transwell migration assays showed the mobility of *OTUB1*-overexpressed cells was markedly increased, whereas that of *OTUB1*-silenced cells was weakened (Fig. 4I–L). Accordingly, the expression of E-cadherin was upregulated and Vimentin was downregulated compared to the control in *OTUB1*-silenced cells (Fig. 4M). Those results revealed that *OTUB1* and *NDUFS2* share the broad congruence in regulating pancreatic cancer cell growth, cell-cycle progression and migration.

### The similarities between *OTUB1* and *NDUFS2* in manipulating mitochondrial membrane dynamics and ATP production in pancreatic cancer cells

**Fig. 5:**
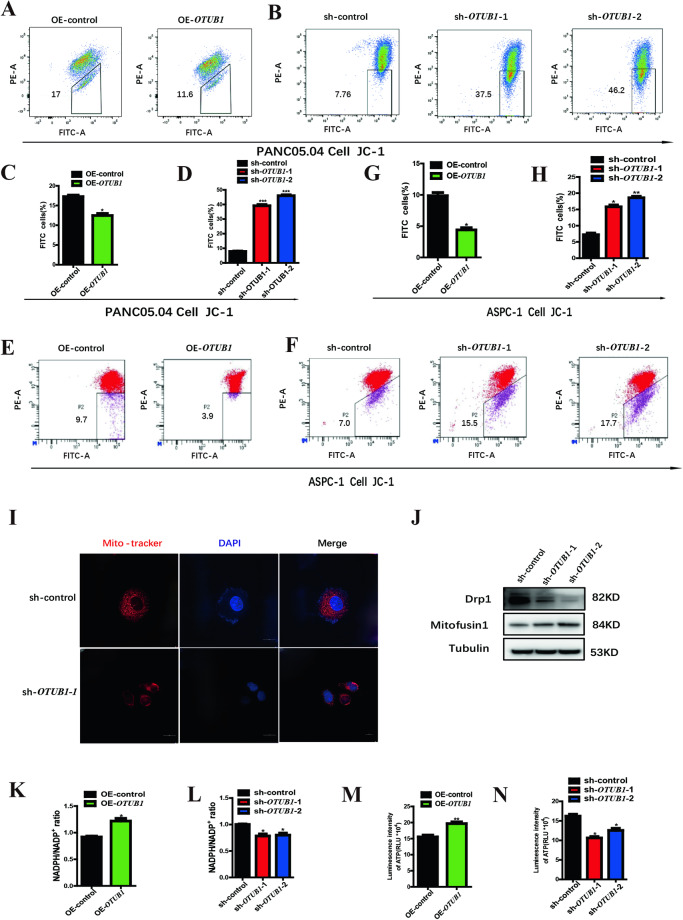
*OTUB1* suppresses mitochondrial death and promotes ATP synthesis in pancreatic cancer cell lines. **A** FACS analysis shows the MMP changes in Panc05.04 cells transfected with OE-*OTUB1* and control plasmids. **B** FACS analysis shows the MMP changes in Panc05.04 cells transfected with sh-*OTUB1* and control plasmids. **C** The statistical result of MMP changes in Panc05.04 cells transfected with OE-*OTUB1* and control plasmids. **D** The statistical result of MMP changes in Panc05.04 cells transfected with sh-*OTUB1* and control plasmids. **E** FACS analysis shows the MMP changes in ASPC-1 cells transfected with OE-*OTUB1* and control plasmids. **F** FACS analysis shows the MMP changes in ASPC-1 cells transfected with sh-*OTUB1* and control plasmids. **G** The statistical result of MMP changes in ASPC-1 cells transfected with OE-*OTUB1* and control plasmids. **H** The statistical result of MMP changes in ASPC-1 cells transfected with sh-*OTUB1* and control plasmids. **I** Mito-Tracker staining shows the changes in mitochondrial morphology in Panc05.04 cell transfected with sh-*OTUB1* and control plasmids. **J** Western blotting shows the expression of Drp1 and Mfn2 in Panc05.04 cells transfected with sh-*OTUB1* and control plasmids. **K** NADP^+^/NADPH ratios in the culture media were measured in Panc05.04 cells transfected with OE-*OTUB1* and control plasmids. **L** NADP^+^/NADPH ratios in the culture media were measured in Panc05.04 cells transfected with sh-*OTUB1* and control plasmids. **M** The ATP concentration in the culture media was measured in Panc05.04 cell transfected with OE-*OTUB1* and control plasmids. **N** The ATP concentration in the culture media was measured in Panc05.04 cell transfected with sh-*OTUB1* and control plasmids.

Given the result of the protein mass spectrometry, we inferred that *OTUB1* may modulate mitochondrial dynamics and functions in the same way as *NDUFS2*. Therefore, MMP was measured by JC-1 in *OTUB1*-overexpressed and -silenced cells, respectively. The result shows that overexpression of *OTUB1* significantly increased the MMP in Panc05.04 cells, and thereby inhibited mitochondrial cell death (Fig. 5A, C). In accordance, knockdown of *OTUB1* markedly declined the MMP, accompanied by aggravated mitochondrial cell death (Fig. 5B, D). Similar results were observed in another pancreatic cancer cell line, Aspc-1 (Fig. 5E–H). In addition, confocal analysis revealed that the mitochondria, stained by Mito-Tracker, in the cytoplasm of *OTUB1*-silenced cells were considerably shorter and fewer than those in the control cells (Fig. 5I). Those results suggested that just like *NDUFS2*, *OTUB1* has a pivotal role in mitochondrial dynamics and cell fate determination in pancreatic cancer cells. We also examined the expression of *Drp1* and *Mfn1* after knockdown of *OTUB1*.The result showed that the expression of Drp1 was downregulated while *Mfn1* was upregulated, which means intracellular mitochondrial division decreased and mitochondrial fusion increased (Fig. 5J). This causes a decrease in the number of mitochondria in the cells. To further validate the function similarity of *OTUB1* on cellular redox function and energy production, the NADPH/NADP^+^ ratio and ATP concentration in the culture media were determined. Overexpression of *OTUB1* increased the NADPH/NADP^+^ ratio and ATP concentration in Panc05.04 cells in contrast to the control (Fig. 5K, M), and knockdown of it decreased the NADPH/NADP^+^ ratio and ATP concentration in accordance, suggesting a major role of *OTUB1* in mitochondrial functions (Fig. 5L, N). Collectively, those data suggested that *OTUB1* behaved much similarly with *NDUFS2* in regulating mitochondrial membrane dynamics and ATP production in pancreatic cancer cells.

### *OTUB1* is a major regulator of *NDUFS2* stability in pancreatic cancer

**Fig. 6:**
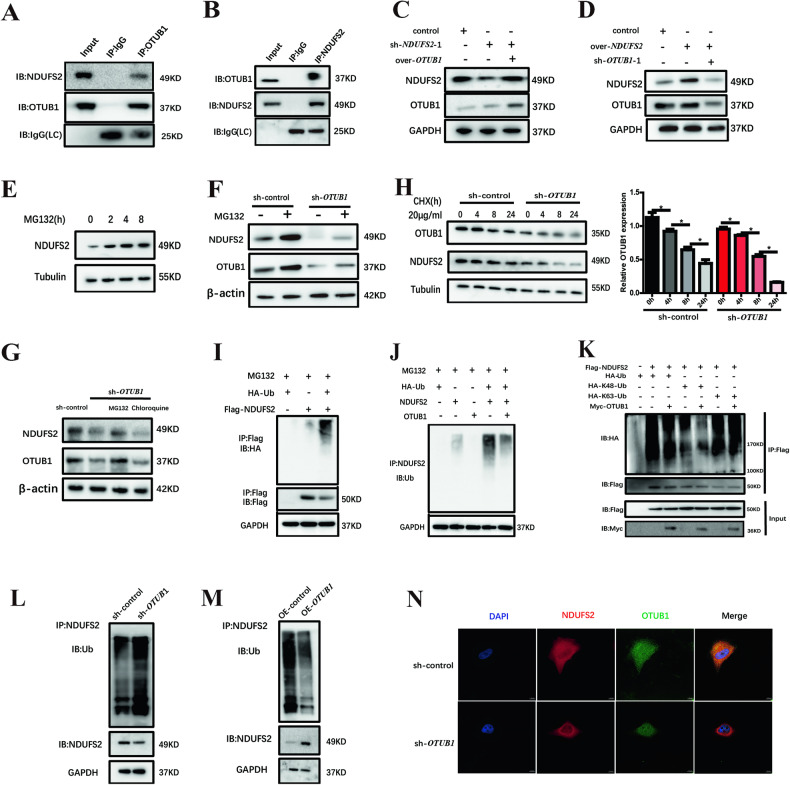
*OTUB1* interacts with *NDUFS2* and rescues *NDUFS2* from proteasomal degradation in pancreatic cancer. **A** *OTUB1* interacted with *NDUFS2* was found via immunoprecipitation assay. **B** *NDUFS2* interacted with *OTUB1* was found via immunoprecipitation assay. **C** Western blotting detecting the expression of *NDUFS2* and *OTUB1* in Panc05.04 cells when co-transfected with sh-*NDUFS2* and OE-*OTUB1* plasmids. **D** Western blotting detected the expression of *NDUFS2* and *OTUB1* when co-transfected Panc05.04 cells with sh-*OTUB1* and OE-*NDUFS2* plasmid. **E** Western blotting detecting the expression of *NDUFS2* in Panc05.04 cells treated with MG132. **F** Western blotting detecting the expression of *NDUFS2* in Panc05.04 cells transfected with sh-*OTUB1* plasmid and treated with MG132. **G** Western blotting detecting the expression of *NDUFS2* in Panc05.04 cells transfected with sh-*OTUB1* plasmid and treated with MG132 and chloroquine. **H** Western blotting detecting the expression of *NDUFS2* and *OTUB1* in Panc05.04 cells transfected sh-*OTUB1* plasmid and treated with CHX. **I** Immunoblotted with the indicated antibodies in Panc05.04 cells immunoprecipitated with FLAG antibody when transfected with HA-*Ub* plasmid and Flag-*NDUFS2* plasmid. **J** Western blotting detecting the expression of *Ub* in Panc05.04 cells immunoprecipitated with *NDUFS2* antibody when transfected with *Ub*, *NDUFS2* and *OTUB1* plasmid. **K** Western blotting detecting the ubiquitination type of *NDUFS2* in Panc05.04 cells. **L** The effect of *OTUB1* knockdown on ubiquitination of NDUFS2 in Panc05.04 cells. **M** The effect of OTUB1 overexpression on ubiquitination of *NDUFS2* in Panc05.04 cells. **N** Immunofluorescence shows the expression of *NDUFS2* and *OTUB1* in Panc05.04 cells transfected with the control and sh-*OTUB1* plasmid.

Giving that the expression of *OTUB1* and *NDUFS2* exhibited tightly correlations in vitro, we presume that *OTUB1* may stabilize *NDUFS2* by direct interaction. Endogenous co-IP assay showed that OTUB1 and NDUFS2 interacted with each other in PANC05.04 cells (Fig. 6A, B). To examine whether the promoting role of *OTUB1* in pancreatic cancer cells was mediated by *NDUFS2*, rescue experiments were performed in Panc05.04 cells. As shown in Fig. 6C, the expression of *NUDFS2* decreased significantly after being transfected with sh-*NDUFS2* plasmid, but these decreases were abolished after co-transfection with OE-*OTUB1* plasmids. Meanwhile, the expression of *NDUFS2* was overexpressed significantly after being transfected with OE-NDUFS2 plasmid, but the overexpression was abolished by co-transfection with sh-*OTUB1* plasmids (Fig. 6D), suggesting that *OTUB1* could promotes the expression of NDUFS2.

To further test that whether *OTUB1* could promote NDUFS2 protein degradation, MG132, chloroquine and CHX assay were used. We found that MG132, which is a proteasome inhibitor, could promote the expression of *NDUFS2* at protein level in a time-dependent manner (Fig. 6E). Moreover, the expression of NDUFS2 was reduced when *OTUB1* was knockdown, and MG132 could reverse this process (Fig. 6F). In contrast, there was no significant changes observed when treated with the lysosomal inhibitor Chloroquine, which means NDUFS2 is not degraded in the lysosomal pathway (Fig. 6G). Additionally, the expression of NDUFS2 was dramatically reduced after *OTUB1* knockdown when treated with protein synthesis inhibitor, CHX (Fig. 6H). These above results suggesting that *OTUB1* may be involved in the ubiquitination degradation of NDUFS2.

The downregulation of *NDUFS2* in *OTUB1*-silenced cells prompt us to test whether *OTUB1* is a potential DUB for NDUFS2. Since ubiquitination of NDUFS2 has not been reported, we tested whether NDUFS2 undergoes ubiquitination modification. As shown in Fig. 6I, NDUFS2 could be notably ubiquitylated when co-transfected with exogenous ubiquitin. Meanwhile, we also found that it could significantly reduce the ubiquitination level of NDUFS2 when co-transfected with OE-*OTUB1* plasmid, suggesting OTUB1 may act as a DUB for NDUFS2 (Fig. 6J). As we known, the lysine 48 (K48)- and lysine 63 (K63)-linked polyubiquitination are the two most abundant types that contribute to the synthesis of polyubiquitin chains on protein substrates. Here, we focused on detecting the changes in the K48- and K63-linked chains which may be involved in NDUFS2 degradation. We found that K48-linked ubiquitin completely abolished polyubiquitin chain formation on NDUFS2 (Fig. 6K), while K63-linked ubiquitin (K63-Ub) showed no obvious effect of polyubiquitin chain on NDUFS2, suggesting that OTUB1 promoted NDUFS2 degradation via K48-linked polyubiquitin chains. To further substantiate our conclusions, we checked the effect of OTUB1 on endogenous ubiquitination of NDUFS2 in pancreatic cancer cells. The results showed that overexpression of *OTUB1* notably decreased the ubiquitination of NDUFS2, and knockdown of *OTUB*1 resulted in the increased the ubiquitination of NDUFS2 (Fig. 6L, M). Immunofluorescence assay (IF) suggested that NDUFS2 and OTUB1 were co-localized in the cytoplasm, and the expression of NDUFS2 was reduced when *OTUB1* was knockdown (Fig. 6N). Taken together, those results indicated that *OTUB1* participated in the proteasomal degradation of NDUFS2.

### *OTUB1* promotes the growth of Panc05.04 cells via increasing *NDUFS2* in vivo

**Fig. 7:**
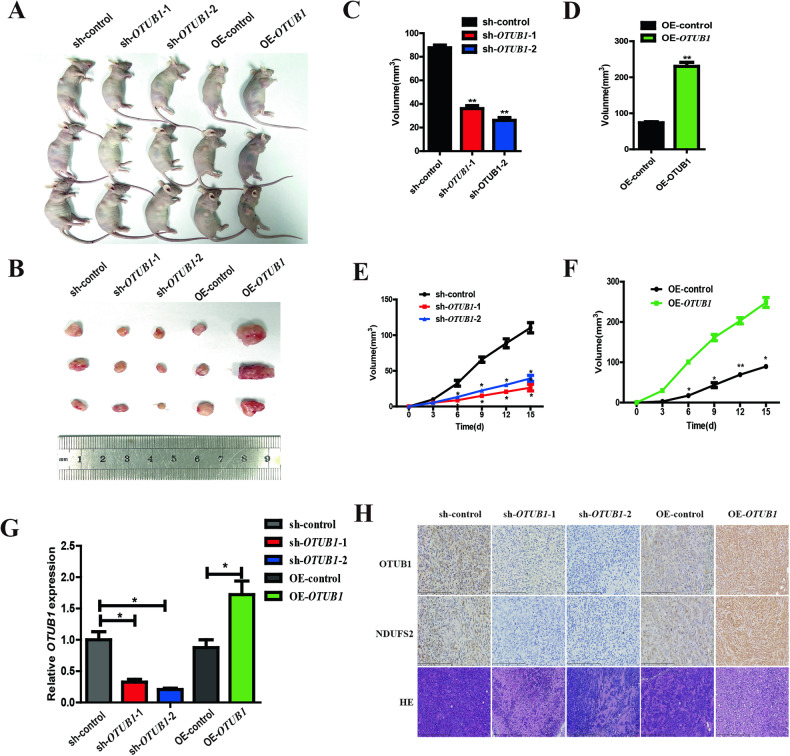
*OTUB1* promotes the growth of Panc05.04 cells via increasing *NDUFS2* in vivo. **A** Panc05.04 cells transfected with OE-*OTUB1*, sh-*OTUB1* and their control plasmids were transplanted subcutaneously in nude mice. **B** Images of OE-*OTUB1*, sh-*OTUB1* and control xenograft tumors isolated from the nude mice. **C** Tumor size was measured in the sh-*OTUB1* and control xenograft groups. **D** Tumor size was measured in the OE-*OTUB1* and control xenograft groups. **E** The tumor volumes were measured every 3 days in sh-*OTUB1* and control xenograft groups. **F** The tumor volumes were measured every 3 days in OE-*OTUB1* and control xenograft groups. **G** qRT-PCR detecting the expression of *OTUB1* and *NDUFS2* in xenograft tumors. **H** Representative images of H&E and IHC staining detecting the expression of OTUB1, NDUFS2 in OE-*OTUB1* and sh-*OTUB1* xenograft tumors from the nude mice.

Previously, we characterized *OTUB1* as a co-factor of *NDUFS2* in the regulation of cell survival, proliferation and mobility in vitro and especially in clinical samples. To further verify the effects of *OTUB1* on tumor formation in vivo, we injected Panc05.04 cells into subcutaneous tissues in nude mice after knockdown and overexpression of *OTUB1*, respectively. The result shows that when *OTUB1* was downregulated, the growth of subcutaneous tumors was significantly decreased compared to the control (Fig. 7A, B, E), and overexpression of *OTUB1* resulted in a significant increase in the volume of subcutaneous tumors compared to the control (Fig. 7A, B, F). Statistical analysis suggested that the growing rate was apparently reduced when *OTUB1* was knockdown (Fig. 7C), while it was apparently increased after overexpression of *OTUB1* (Fig. 7D). qRT-PCR was used to verify the expression of OTUB1 in each group **(**Fig. 7G). Additionally, the result of IHC staining suggested that the expression of NDUFS2 was increased in the *OTUB1*-overexpressed group, and it was reduced in the *OTUB1*-silenced group (Fig. 7H). The results are consistent with previous IF results.

## Discussion

Pancreatic cancer is the fourth leading causes of cancer death in the USA and leads to an estimated 227 000 deaths per year worldwide [22]. Surgical resection at present offers the only chance of cure, but only about 4% of patients will live 5 years after diagnosis [23]. Since pancreatic cancer responds poorly to most chemotherapeutic agents, thus there is an urgent need to find new therapeutic approaches and targets.

Mitochondria, the main sites of oxidative phosphorylation and ATP biosynthesis, provide the majority of the energy required by aerobic cells for maintaining their physiological activities [24, 25]. Targeting mitochondria to manipulate cell death holds tremendous therapeutic potential across different diseases, including various kinds of cancers [26]. In this study, we found that *NDUFS2* plays a critical role in the proliferation, cell-cycle precession, colony formation and migration of pancreatic cancer cells. It was further verified that *NDUFS2* promoted pancreatic cancer cell migration by regulating the expression of E-cadherin and vimentin. NDUFS2 perhaps affecting mitochondrial dynamics and functions in pancreatic cancer cells by altering MMP. Recently, *NDUFS2* has been identified as a redox-sensitive oxygen sensor [27]. We verified that interfering with *NDUFS2* inhibited intracellular ATP synthesis and decreased the NADPH/NADP^+^ ratio significantly which means NDUFS2 could play an important role in the ATP synthesis and redox system. In addition, we confirmed that the levels of NDUFS2 and OTUB1 were positively correlated through Co-IP, both of which were abundantly expressed in pancreatic cancer.

OTUB1 is one of the most highly expressed DUBs in cells [28]. DUBs are considered as potential novel therapeutic targets for various diseases [13]. O*TUB1* is overexpressed in a wide variety of human tumors, such as colon cancer, gastric cancer, bladder and breast cancer [29–33]. However, the functions of *OTUB1* in pancreatic cancer cells is rarely reported. In this report, we found that *OTUB1* phenocopied the function of NDUFS2. Co-IP assay and western blot assays further revealed that *OTUB1* increased the protein stability of *NDUFS2* by deubiquitylation. Taking together, our present study revealed that *OTUB1* played a crucial role in the development of pancreatic cancer, and NDUFS2, identified as a substrate of *OTUB1*, might be responsible for the observed phenotypes in the absence of *OTUB1* in pancreatic cancer.

A recent report showed that *OTUB1* may accelerate metastasis of pancreatic cancer by inhibiting *FOXM1* degradation [20]. Here, we showed that *NDUFS2* inactivation inhibited tumor growth in a mouse xenograft pancreatic cancer model, implicating a potential role of *NDUFS2* in the development of pancreatic cancer in vivo. Treatment targeting *NDUFS2* may provide us novel approaches for the treatment of pancreatic cancer. Due to the widespread distribution and high cellular expression of *OTUB1* and *NDUFS2* in tissues, it may be difficult to inhibit their expression in clinic, but treatment targeting them may provide significant benefit to patients diagnosed with multiple tumors [34].

In summary, our study provides new insights into the biological function of *OTUB1* and *NDUFS2*, indicating that targeted therapy against *OTUB1* and/ or *NDUFS2* could hold the premise to provide a new therapeutic method for the treatment of pancreatic cancer, but the specific mechanism by which the *OTUB1*/*NDUFS2* axis regulates the mitochondrial death of pancreatic cancer cells needs to be further experimentally investigated and consolidated.

## Material and methods

### Cell lines and culture

The human pancreatic cancer ASPC-1 cell was cultured in RPMI-1640 (31870082; Gibco) supplemented with 10% FBS (16140071; Gibco) and 100X Penicillin-Streptomycin Solution (SV30010,hyclone), PANC05.04 cells were cultured in F12k media (#21127022, Gibco) supplemented with 10% FBS and 100× Penicillin–Streptomycin Solution. Cells were incubated in a humidified incubator at 37 °C with 5% CO_2_.

### Cell transfection

sh-*OTUB1*, OE-*OTUB1*, sh-*NDUFS2*, OE-*NDUFS2* and their each control plasmids s were purchased from Genechem (Shanghai, China) company. Transient transfections were performed with jetPRIME (#150-15, Polyplus, France) transfection reagent following the manufacturer’s instructions. The sequence of sh-NDUFS2-1 was 5′-GCAGATGTCGTTGCCATCATA-3′; the sequence of sh-NDUFS2-2 was 5′-GCTGTTATGTACCCAAGCAAA-3′. The sequence of sh-OTUB1-1 was 5′-GCAAGUUCUUCGAGCACUU-3′; the sequence of sh-OTUB1-2 was 5′-GCGACUCCGAAGGUGUUAATT′.

### Cell proliferation assay

Cell viability was quantified using the Cell Counting Kit-8 assay(B34304, Selleckchem). Cells were seeded into 96-well plates at a density of 3 × 10^3^ cells/well and were cultured in a humidified atmosphere with 5% CO_2_ at 37 °C. Subsequently, 10 μl CCK-8 reagent was added to each well. The absorbance was measured at 450 nm after 2 h. The CCK-8 assay was performed every 24 h for 4 consecutive days.

### Colony formation assay

For colony formation analysis, 1000–1500 cells per well were plated in six-well plates and allowed to grow for 14 days after the indicated treatments, then we fixed the cells with 4% paraformaldehyde (P1110, Solarbio), and stained the cells with 5% crystal violet (G1063,Solarbio).

### Cell-cycle assay

For the cell-cycle assay, the Cell-Cycle Staining Kit (CCS012, Multi Sciences Biotech Co., Ltd) was used according to the manufacturer’s protocol. Briefly, cells were harvested and fixed in 70% ethanol at 4 °C overnight. Cells were then treated with 1 ml DNA staining solution and 10 μl permeabilization at 37 °C in the dark for 30 min. Cells were then analyzed via flow cytometry.

### Transwell migration assay

A total of 2 × 10^5^ cells which were suspended in 1.5 ml serum-free culture media were added to the top chamber of 6-well transwell plates (3428, Corning), and 2.6 ml culture media containing 10% FBS was added to the bottom chamber. After incubating at 37 °C for 48 h, the chambers were washed with PBS twice, and these cells which migrated to the bottom chambers were fixed with paraformaldehyde and stained with crystal violet. Then the number of transitional cells in all chambers was calculated in the 5 visual fields.

### Wound healing assay

Cells were seeded on 6-well plate and grew to the pavement overnight. After 24 h of transfection, a channel was drawn on the monolayer cells with 10 μl micropipette tip. Then cells were washed with PBS twice and cultured in serum-free culture media at 5% CO_2_, 37 °C for an additional 24 h. Photographs were taken by an inverted Leica phase contrast microscope at 0 h and 24 h.

### Measurement of mitochondrial membrane potential

To measure mitochondrial membrane potential (MMP), cells were washed with PBS and incubated with JC-1 (C2005, Beyotime Biotechnology) at 37 °C for 20 min in the dark. After incubation with the dye, the plates were washed three times with PBS. Fluorescence was measured first at excitation/emission 485/580 nm (red) and then at excitation/emission 485/530 nm (green) using a flow cytometer (BD, Fortessa). The results were analyzed with FlowJo software.

### Analysis of mitochondrial activity

Cells were plated on poly-l-lysine-coated glass coverslips. Afterward, we stained the cells with 100 nM Mito-Tracker(C1048, Beyotime Biotechnology)Deep Red/Green staining solution in the dark for 30 min to visualize mitochondria, followed by confocal imaging (ZEISS, Germany).

### NADP^+^/NADPH and ATP assay

For measurements of NADP^+^/NADPH ratio and ATP concentration, cells were harvested and treated with appropriate buffers as indicated by the providers. In particular, NADP^+^/NADPH ratios were determined using a NADP^+^/NADPH Assay kit (S0179, Beyotime) and were detected using a colorimetric assay under a microplate reader (Feyond-A400, ALLSHENG, China) with detection wavelengths of 570 nm, respectively. The ATP concentration was calculated using an enhanced ATP assay kit (S0027, Beyotime) by measuring chemiluminescence with a luminometer plate reader.

### Western blotting

Cells were harvested 48 h after transfection of the plasmid for immunoblotting analysis.Cells were lysed using RIPA buffer (P0013B, Beyotime Biotechnology). Subsequently, total protein was separated using SDS-PAGE and transferred to a PVDF membrane. Membranes were blocked and were subsequently incubated at 4 °C overnight with the following primary antibodies against: GAPDH (2118, 1:1000; CST), OTUB1 (270959, 1/1000; Abcam),beta-Tubulin (16305, 1:2000; CST), NDUFS2 (sc-390596, 1:1000; MDL Biological, Inc.), E-cadherin(ab233611, 1/10,000; Abcam),Vimentin (ab92547, 1/2000; Abcam); Drp1 (Cell Signaling, 1:1000, 8570S); Mfn2 (Cell Signaling, 1:1000,11925S);HA-Ub(CST,3936S); HA-K48-Ub(CST,8081S); HA-K63-Ub(CST,5261S). All antibodies were used according to the manufacturer’s protocol. Following primary incubation, membranes were incubated with secondary antibodies, HRP-conjugated goat anti-rabbit IgG (PR30011, 1:20000; Proteintech Co., Ltd.) and HRP-conjugated goat anti-mouse IgG (PR30012, 1:20000; Proteintech Co., Ltd.) at room temperature for 1 h. Proteins were visualized using ECL Reagent (42029053, Millipore Sigma). All the statistical results of western blotting were shown in FigureS which is qualified through ImageJ (NIH, USA).

### Drugs and inhibitors

10 μM MG132(HY-13259, MCE) which is a potent proteasome inhibitor and 200 μM Chloroquine (HY-17589A, MCE) which is a lysosomal enzyme inhibitor, was used to treat pancreatic cell lines for 8 h; 20 μg/ml CHX (HY-12320, MCE), which is an inhibitor of protein synthesis, was used to inhibit the protein synthesis at different time.

### Immunofluorescence staining and co-immunoprecipitation

Slides containing human pancreatic tumor tissues and adjacent normal pancreatic tissues were used in our experiment. The slides were incubated with primary antibodies at 4 °C overnight. The following day, after incubation with the corresponding secondary antibody, the nuclei were stained with DAPI (Yeason,40728ES03) before observing by confocal laser microscopy. Co-immunoprecipitation analysis was carried out using the Pierce Co-Immunoprecipitation Kit (88804; Thermo Fisher) according to the manufacturer’s instructions. primary antibodies against: Ub (3936s, 1:1000; CST), IgG light chain (SA00001-7L; 1:1000; Proteintech, Inc.),Flag(ABT2010,1:1000,Abbkine) were used according to the manufacturer’s protocol.

### Immunochemical (IHC)-staining

IHC analyses were done by the Mango Bioservice company (Beijing, China) using primary antibodies against OTUB1, and NDUFS2 at 1:100. The expression levels of OTUB1 and NDUFS2 were assessed using the H score approach as described previously [11].

### Patients and tissue handling

Between September 2022 and June 2023, 8 pairs of PAAD tissues along with neighboring non-tumorous pancreatic tissues were acquired from the Department of Hepatobiliary surgery of Xuanwu hospital during surgery and we obtained informed consent from each subject. The protein from pancreatic cancer and adjacent tissue specimens from patients were extracted using ExKine™ Pro Total Protein Extraction Kit(Abbkine,KTP3007).The rest samples were preserved in 4% paraformaldehyde solution waiting for the IHC and IF staining.

### Animal Studies

Panc05.04 cell line was used for xenograft tumor model. Fifteen five-week-old male nude mice (SPF Bio-Technology Co. Ltd, Beijing) were randomized and blinded divided into five groups (sh-control, sh-*OTUB1*-1, sh-*OTUB1*-2, OE-control and OE-*OTUB1* group; 3 nude mice in each group). Cells were injected subcutaneously with 1 × 10^7^ cells suspended in 100 μl PBS and mixed with 100 μl Matrigel (356231, BD Biosciences, America). These cells were implanted subcutaneously into the dorsal flank sides of the mice. Once the diameter of tumors reached nearly 2 mm, the volume of tumor was measured daily for 2 weeks. The lengths and widths of the tumors were measured every 3 days. Tumor volume (mm^3^) was calculated as length × (width)^2^ × 0.52. On the 15th day, these mice were killed and tumors were extracted and measured. The tumors were fixated with paraformaldehyde, then immunohistochemistry (IHC) staining was performed for *OTUB1*, *NDUFS2*, and HE. All procedures involving mice were approved by the University Committee on Use and Care of Animals at the Capital Medical University and met all regulatory standards.

### Real-time PCR

The mRNA levels of *OTUB1*, and *NDUFS2* in xenograft tumors were determined using qRT-PCR analysis. Total RNA was extracted from cultured cells and 1 mg of total RNA was converted to cDNA using the Revert Aid RT reverse transcription kit (#K1691, Thermo Fisher Scientific). Real-time PCR was done using the SYBR-green premix (#RR600/ 601, Takara) on a Studio Q3 real-time PCR system (Life Technologies). Relative gene expression was determined using the comparative Ct approach and normalized to β-actin. Sequences of the primers are given in Table 2.

**Table 2:** The primer sequences of different genes.

Gene	Forward primer	Reverse primer
β-actin	Forward 5′GACATGCCGCCTGGAGAAAC3′	Reverse 5’AGCCCAGGATGCCCTTTAGT3′
OTUB1	Forward 5′ACAGAAGATCAAGGACCTCCA3′	Reverse 5′CAACTCCTTGCTGTCATCCA3′
NDUFS2	Forward 5′GTCCGATTGCCGATTCAGC3′	Reverse 5′ GCTTGGGTACATAACAGCTCC3′

### Bioinformatics data

Differential expression data of *OTUB1* and all the dysregulated genes in PAAD were obtained from TCGA (https://portal.gdc.cancer.gov/) database. The transcriptional expression of *OTUB1* and *NDUFS2* were extracted from Gepia2(http://gepia.cancer-pku.cn/) database.

### Statistical Analysis

Data analyses were carried out using SPSS 21.0 (IBM Corp., USA) as well as GraphPad Prism 5 (GraphPad Software, CA). All results were from three independent experiments. Normally distributed data were given as mean ± SEM. All tests are two-sided. Statistical comparisons between groups were assessed using the Student’s *T*-test (two-tailed) and ANOVA analysis. Statistical significance was denoted by *P* < 0.05 (*p < 0.05; **p < 0.01; ***p < 0.001).

### Supplementary information

FigureS legend

FigureS

WB

## Data Availability

The published article includes all data sets generated/analyzed for this study.
