# Supplementary material for: OTUB1/NDUFS2 axis promotes pancreatic tumorigenesis through protecting against mitochondrial cell death
Source: Cell Death Discov. 2024 Apr 23;10:190. doi: 10.1038/s41420-024-01948-x (PMC11039712; doi:10.1038/s41420-024-01948-x)
Supplement: Supplementary file 1 — FigureS legend [file 41420_2024_1948_MOESM1_ESM.docx]

**Figure S** The quantification of western blot in Fig1-Fig5 and the IF staining of the other two samples
