# Supplementary figures and images for: OTUB1/NDUFS2 axis promotes pancreatic tumorigenesis through protecting against mitochondrial cell death
Source: Cell Death Discov. 2024 Apr 23;10:190. doi: 10.1038/s41420-024-01948-x (PMC11039712; doi:10.1038/s41420-024-01948-x)

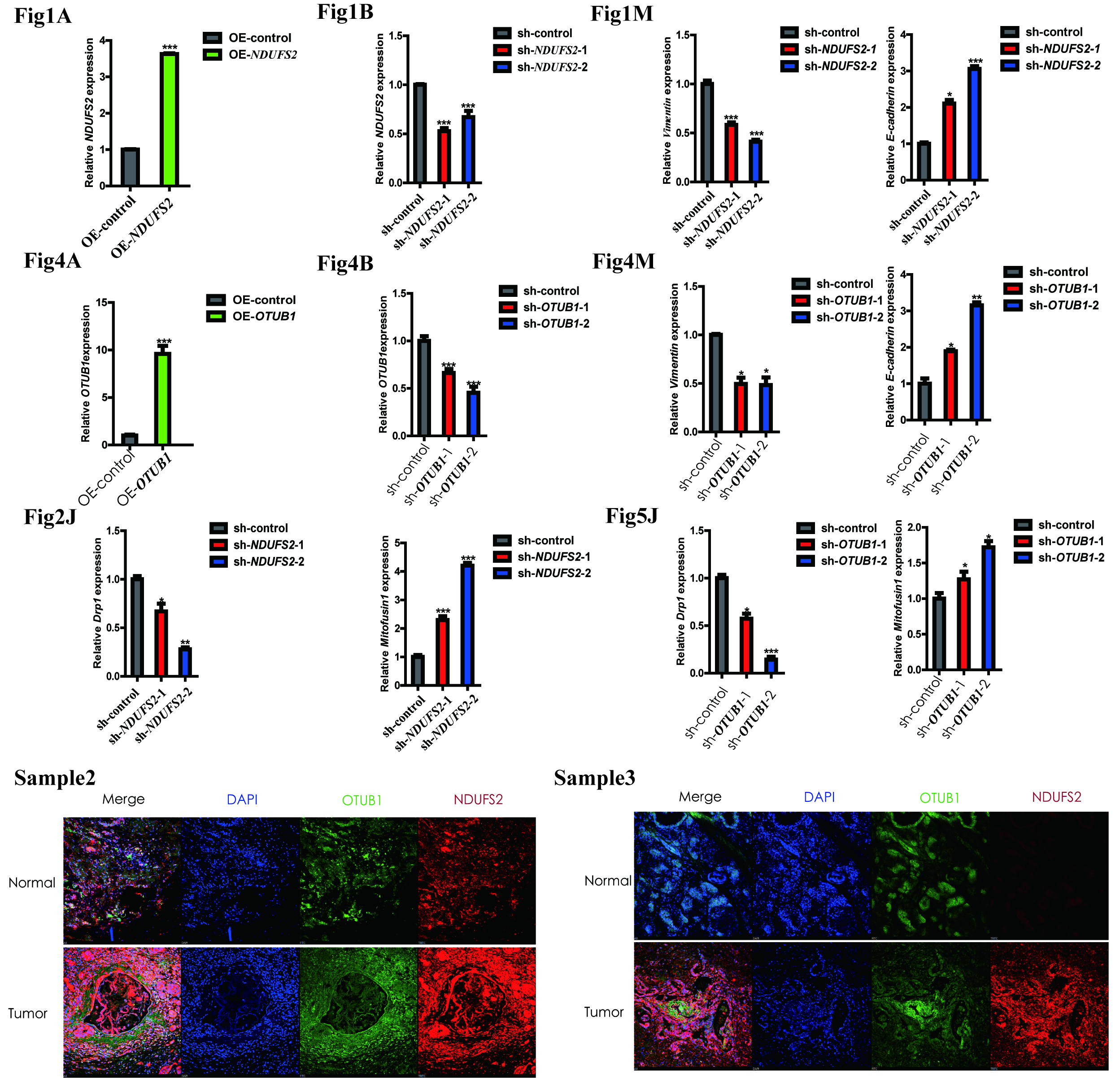

Supplement: Supplementary file 2 — FigureS [file 41420_2024_1948_MOESM2_ESM.tif]
