# Supplementary material for: OTUB1/NDUFS2 axis promotes pancreatic tumorigenesis through protecting against mitochondrial cell death
Source: Cell Death Discov. 2024 Apr 23;10:190. doi: 10.1038/s41420-024-01948-x (PMC11039712; doi:10.1038/s41420-024-01948-x)

**Figure1**

Figure1A left panel


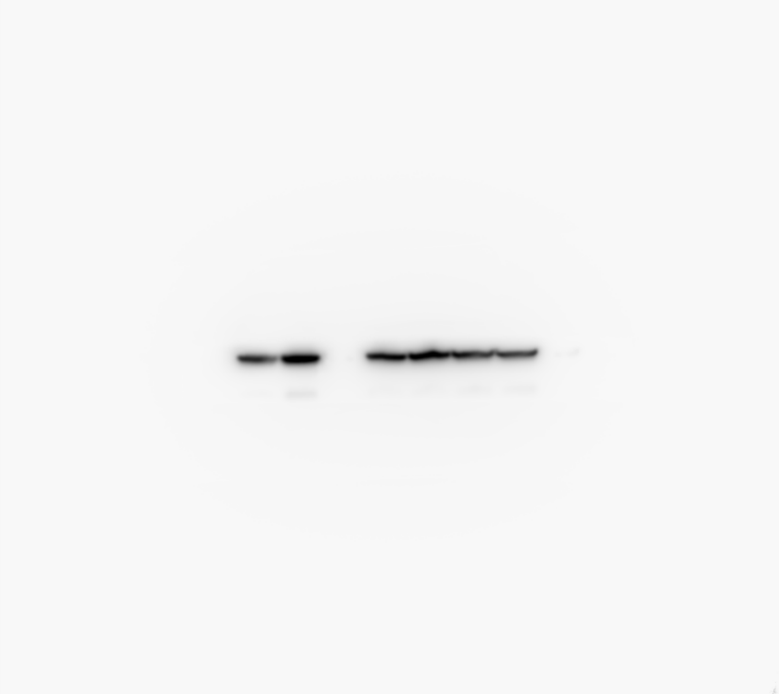


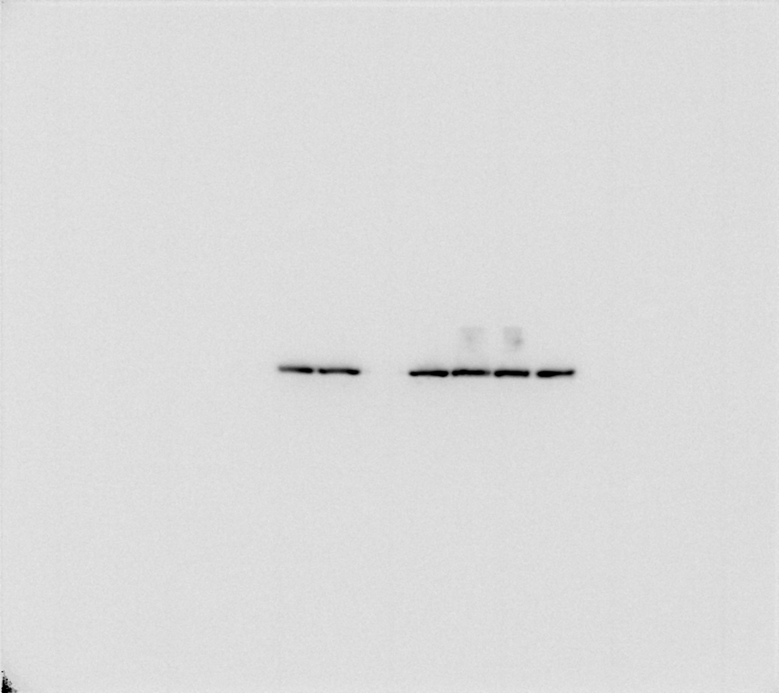


Figure 1B


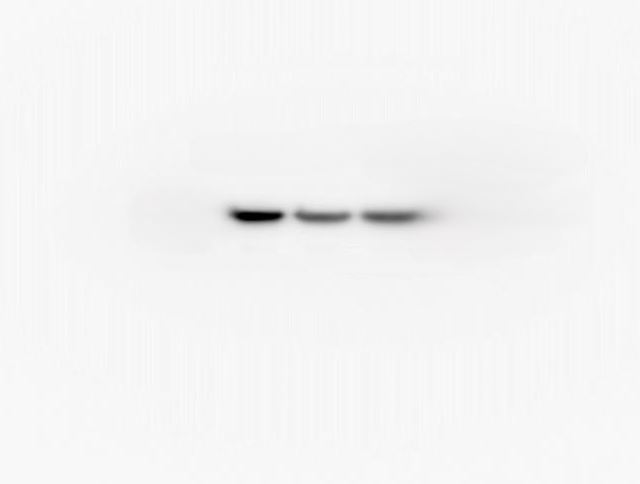





Figure 1M


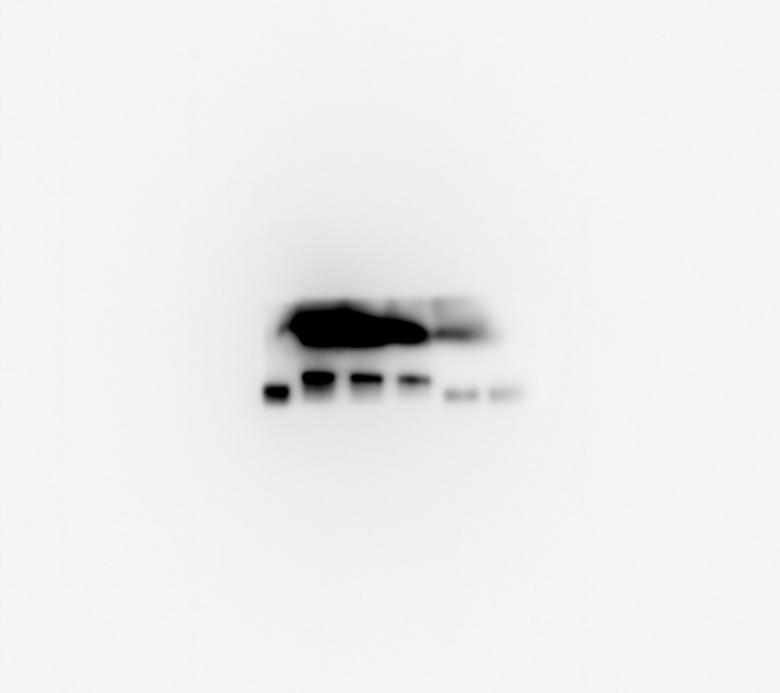

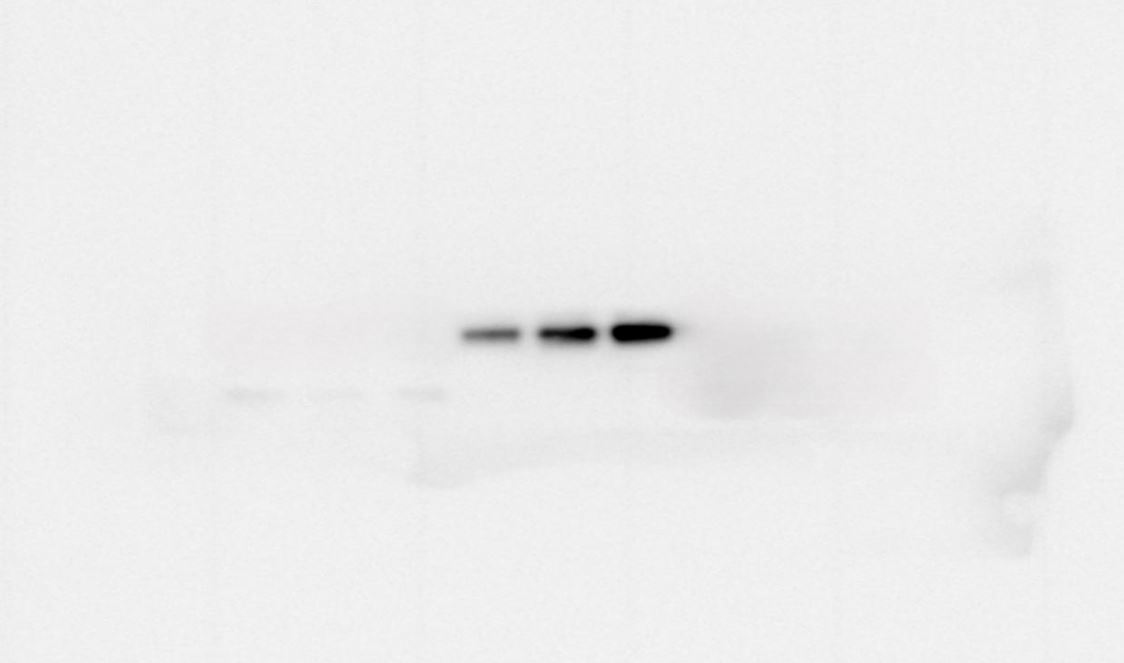

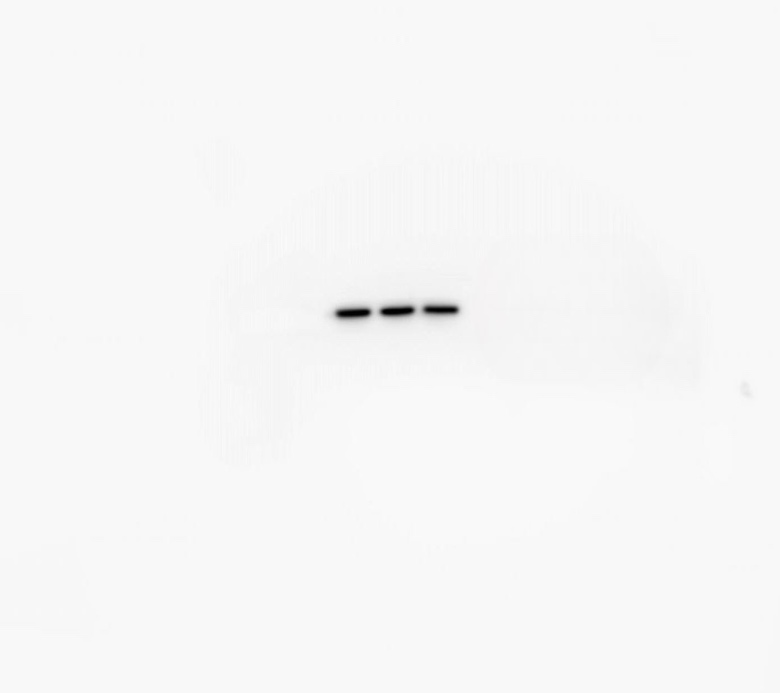


**Figure2**

Figure2J

Row6-8


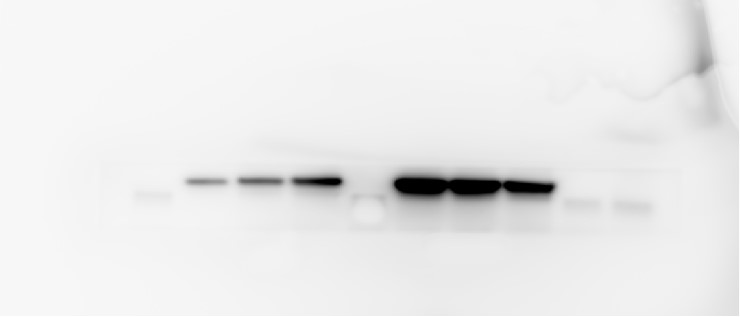


Left panel

l
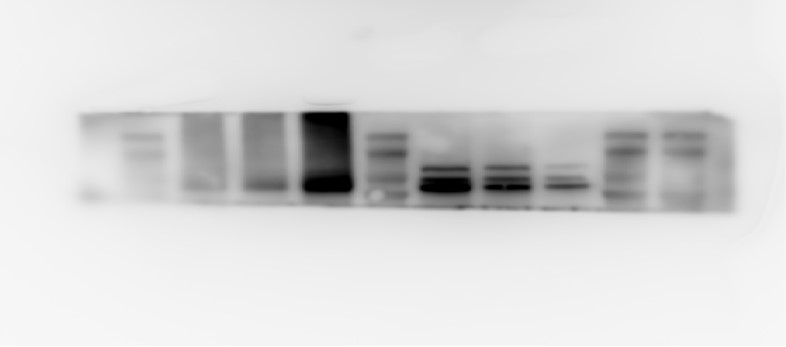


Left panel


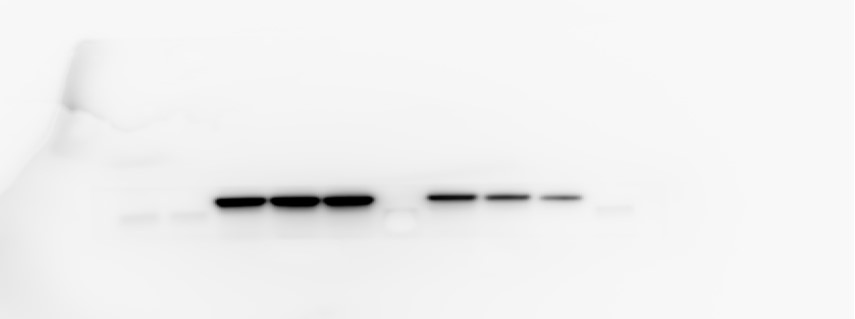


**Figure3**

Figure 3F


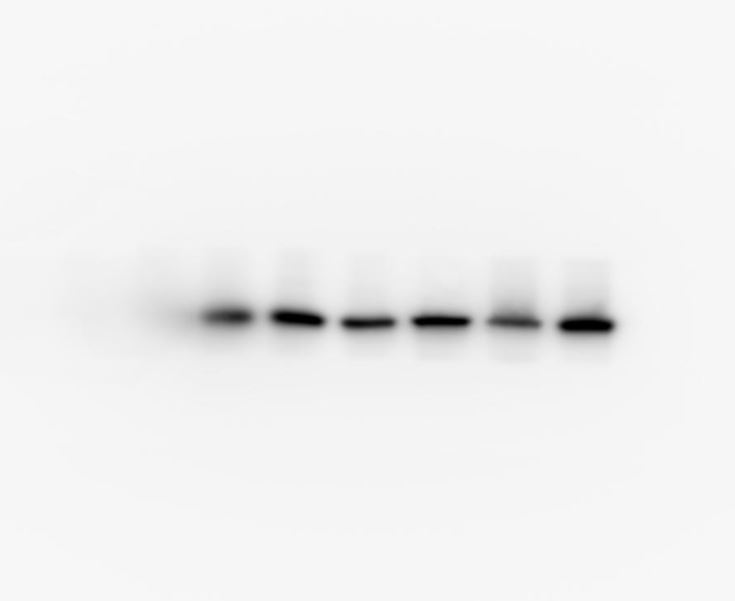


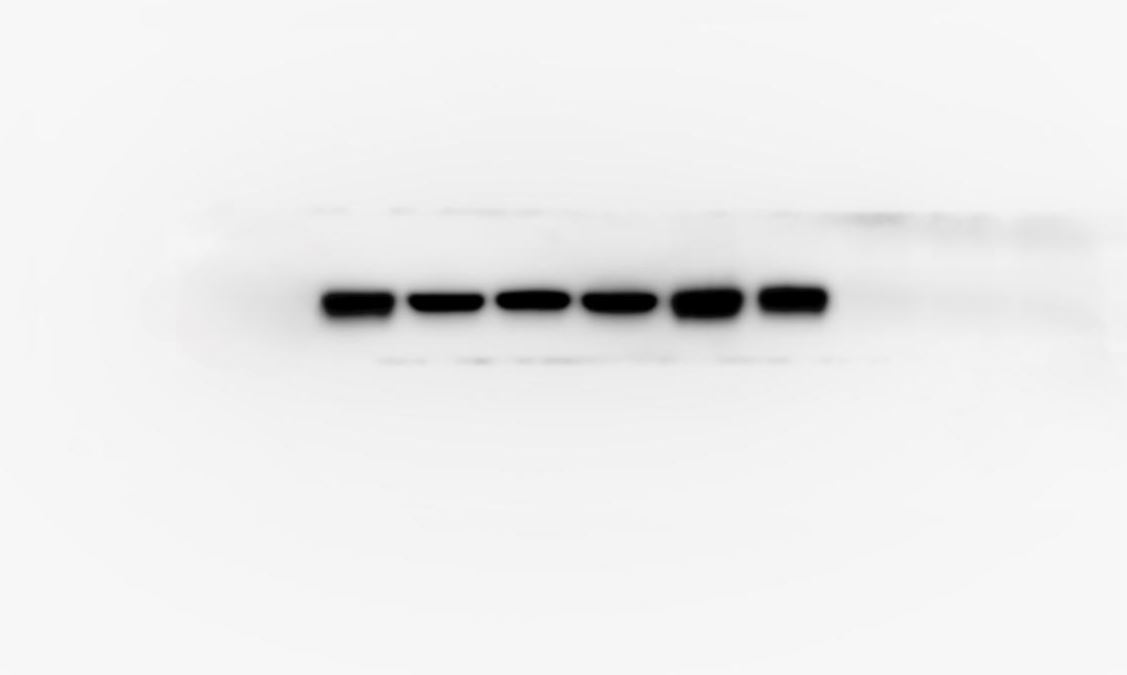


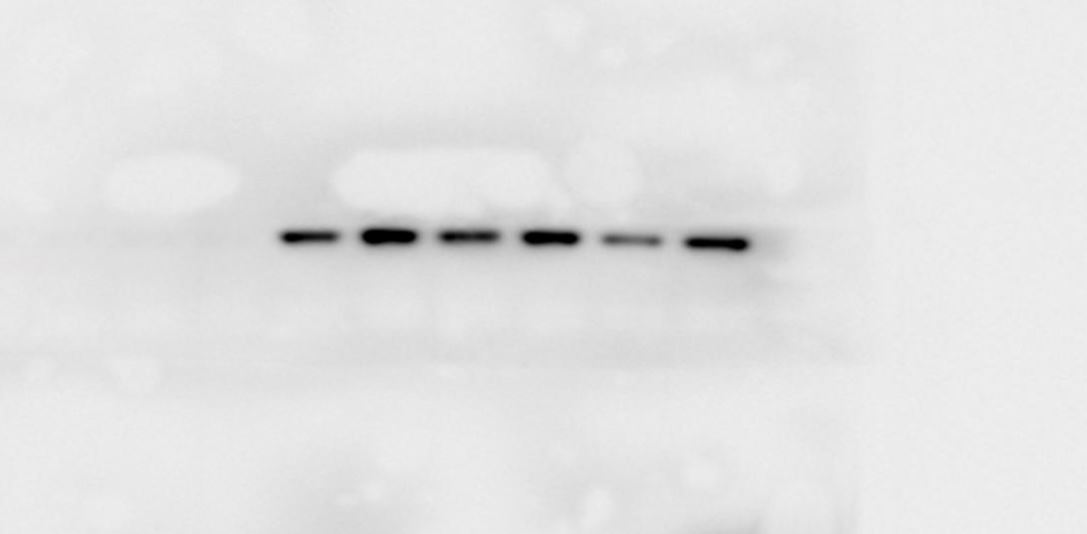


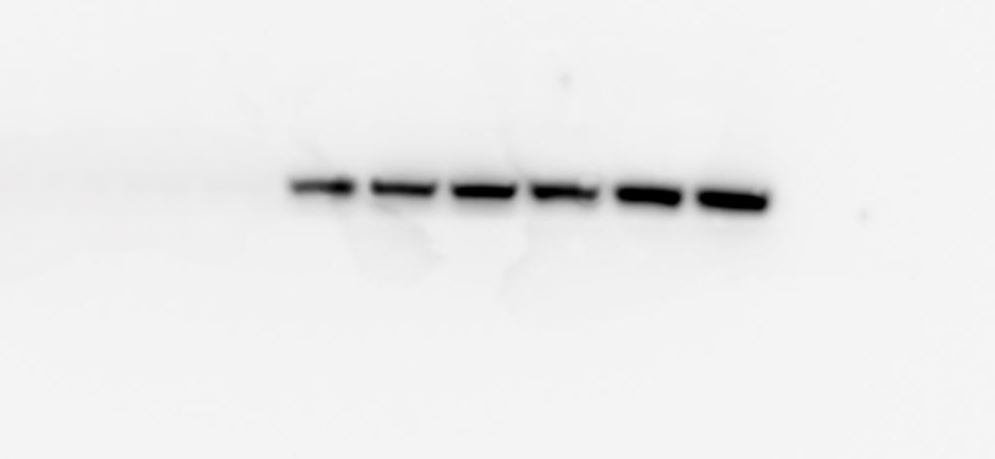


Figure 3H


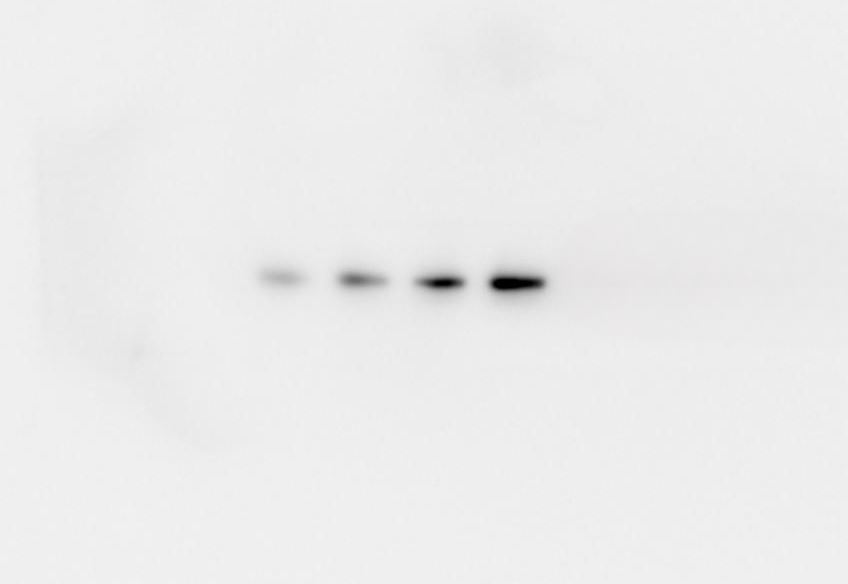


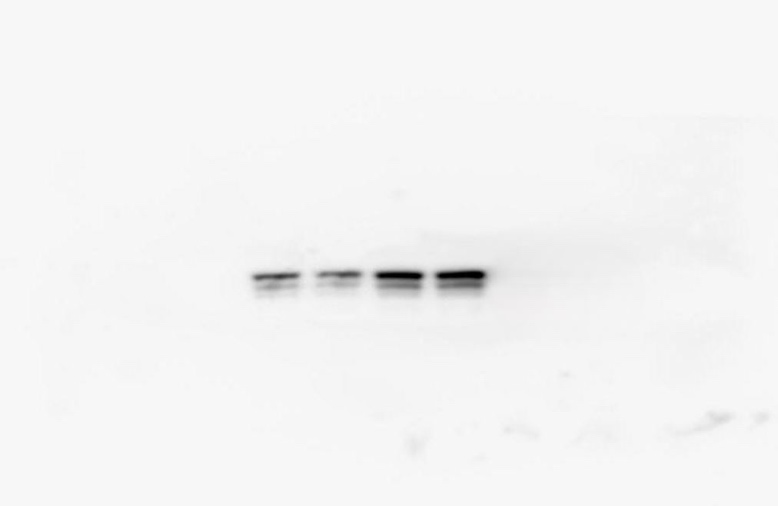


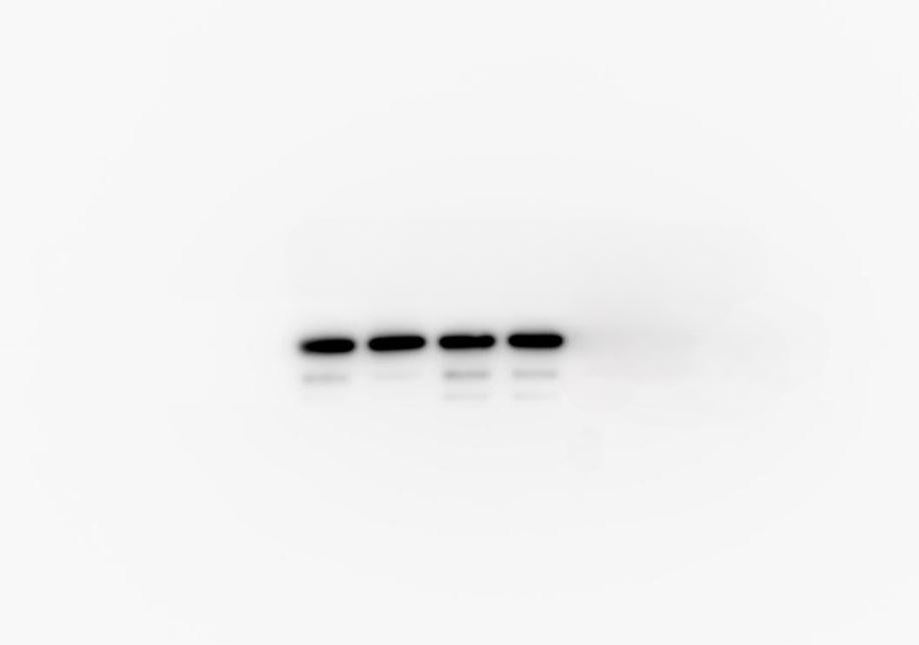


Figure 3I right panel


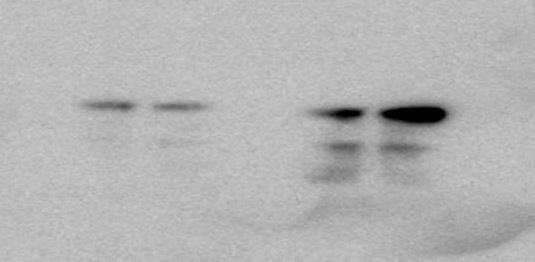


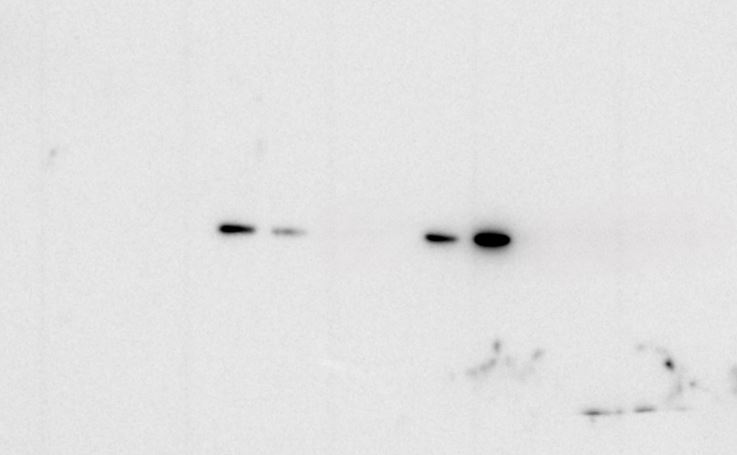


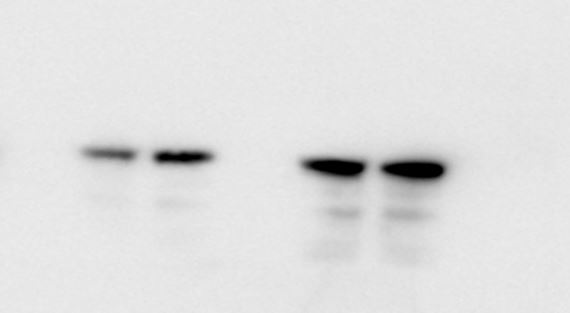


Figure 3J


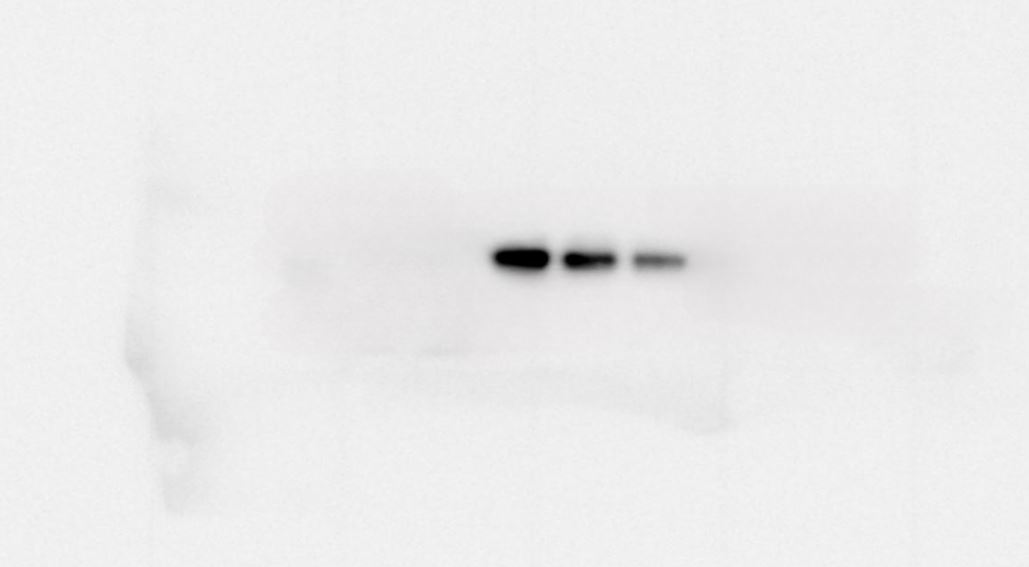


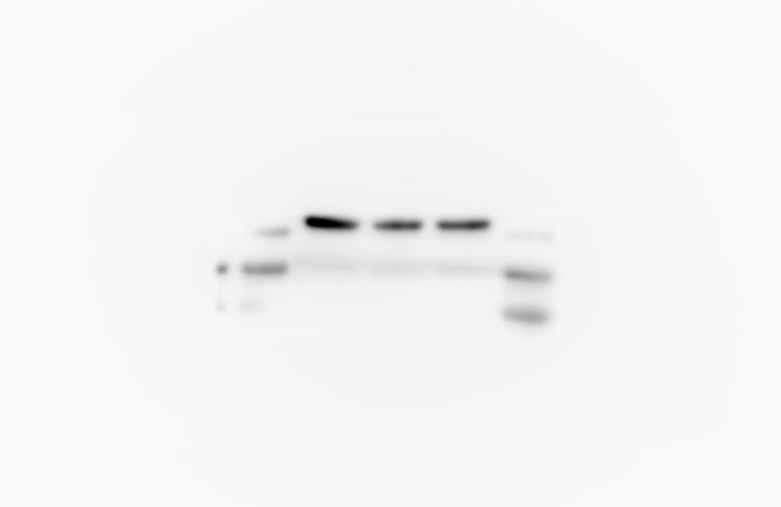


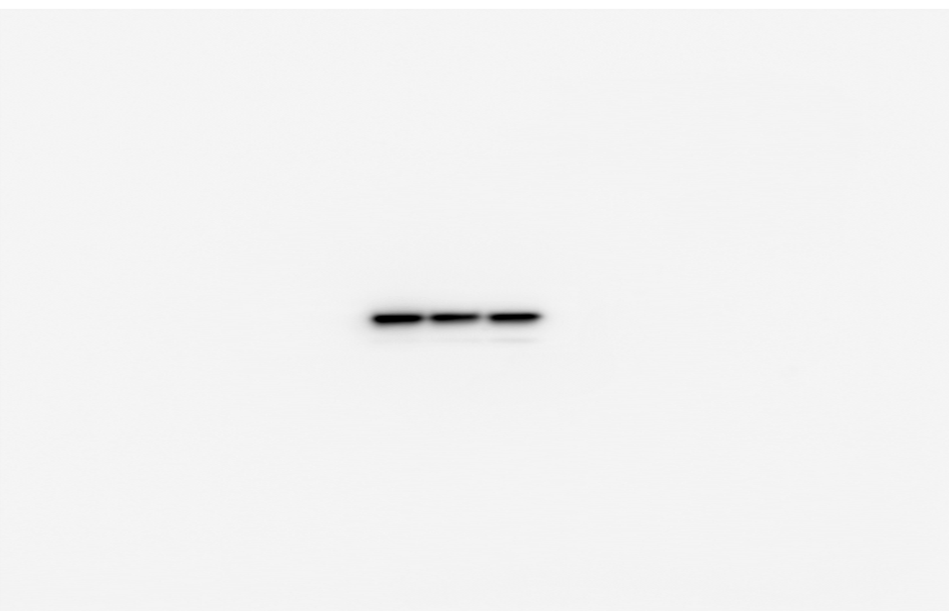


Figure 3K


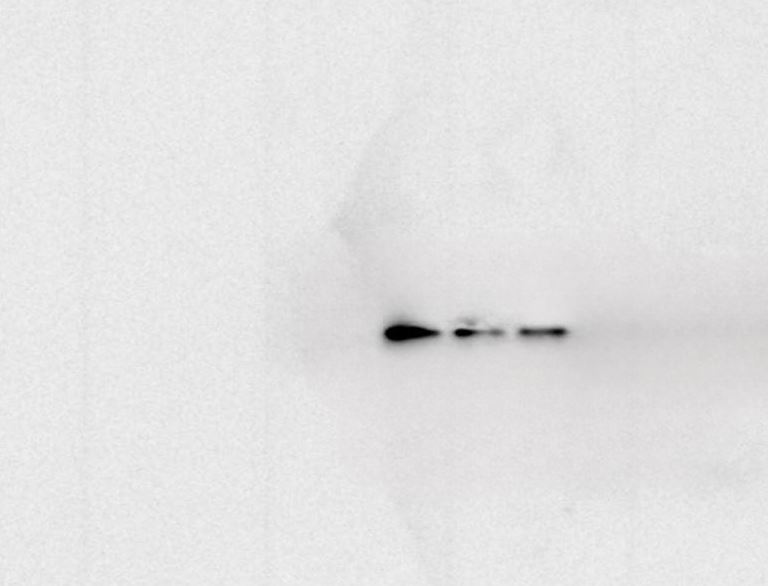


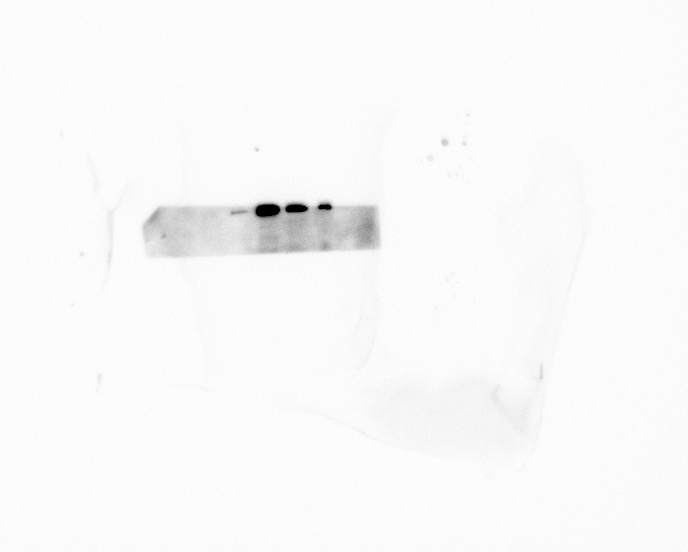


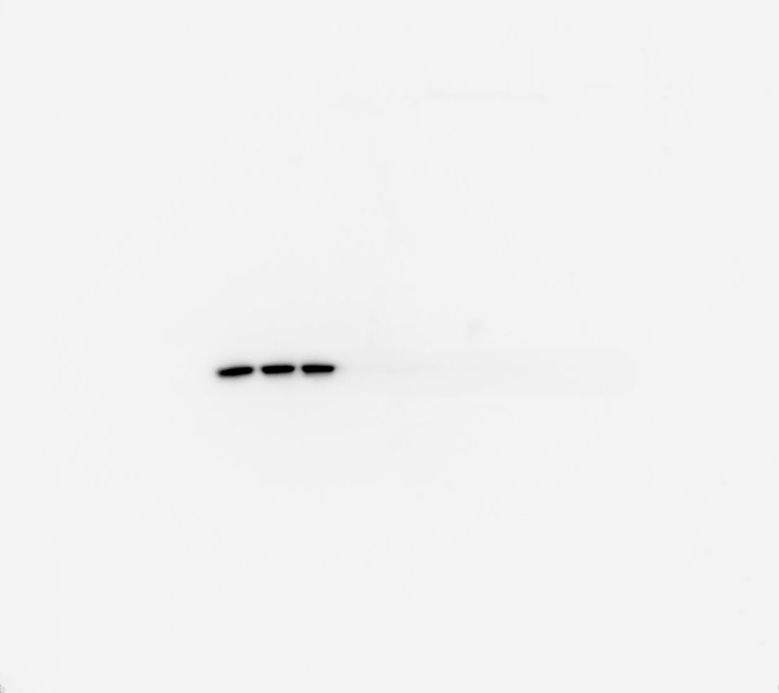


**Figure4**

Figure 4A


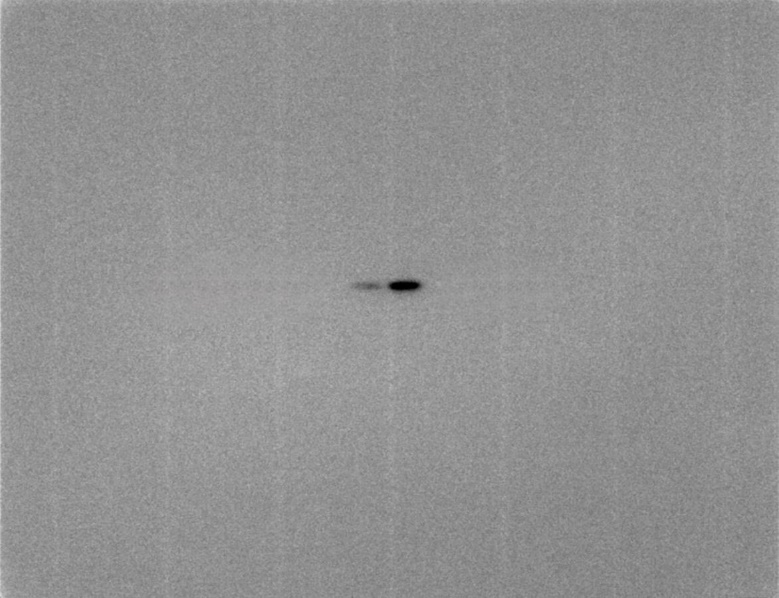


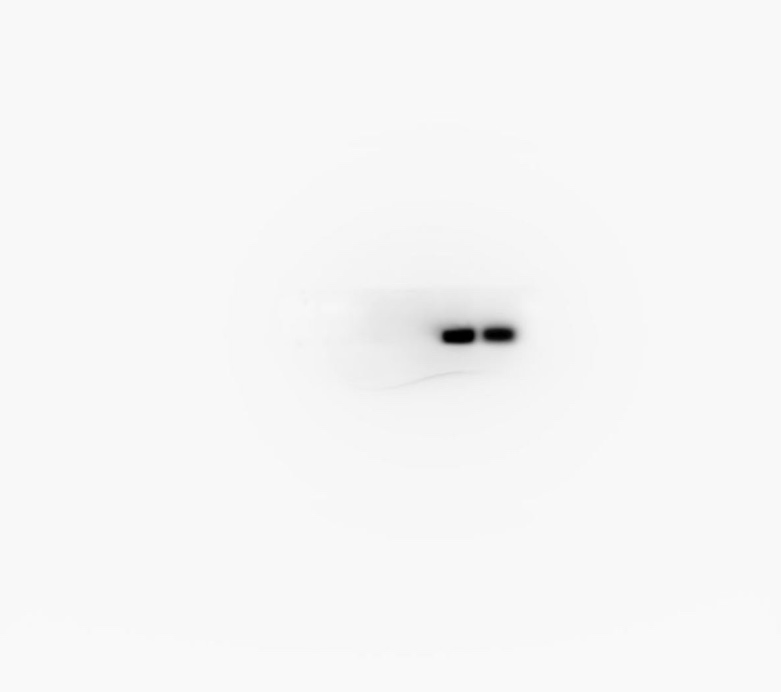


Figure 4B


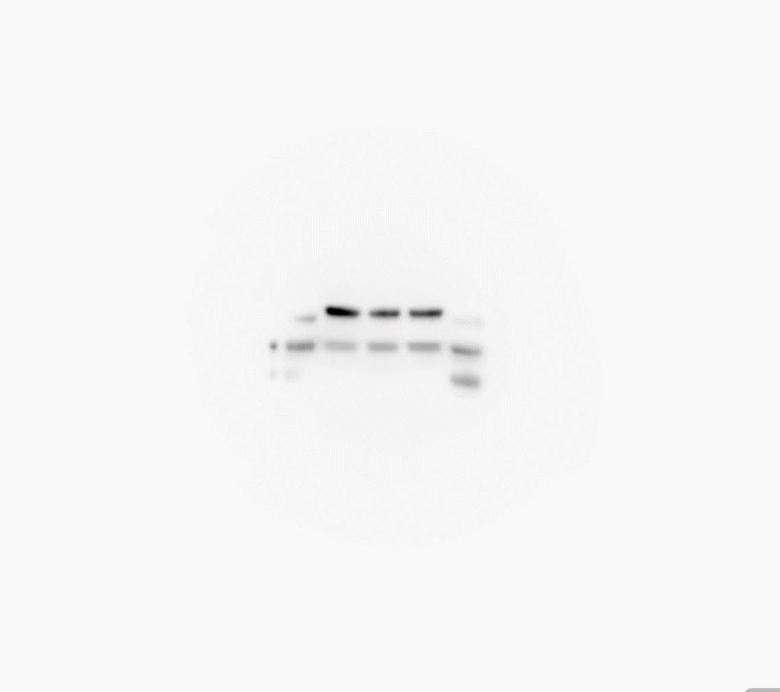


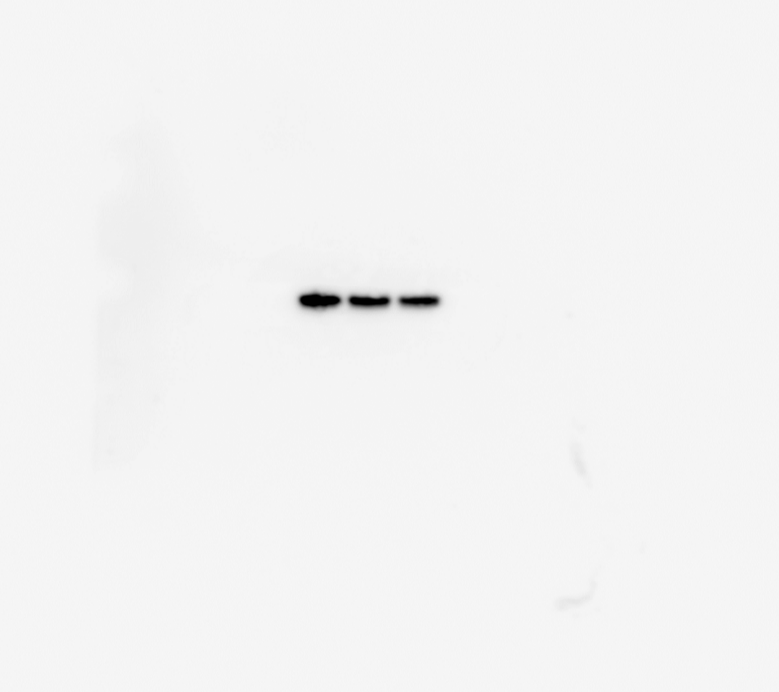


Figure 4M


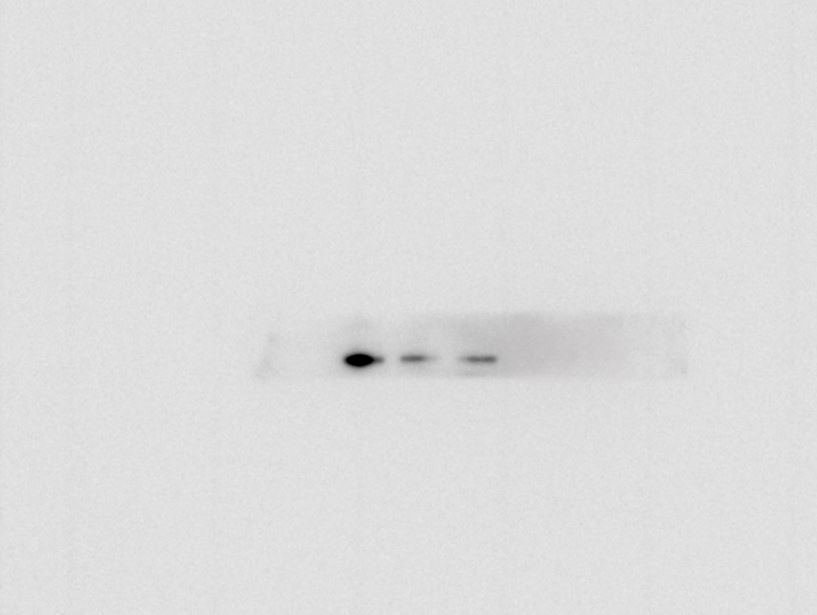


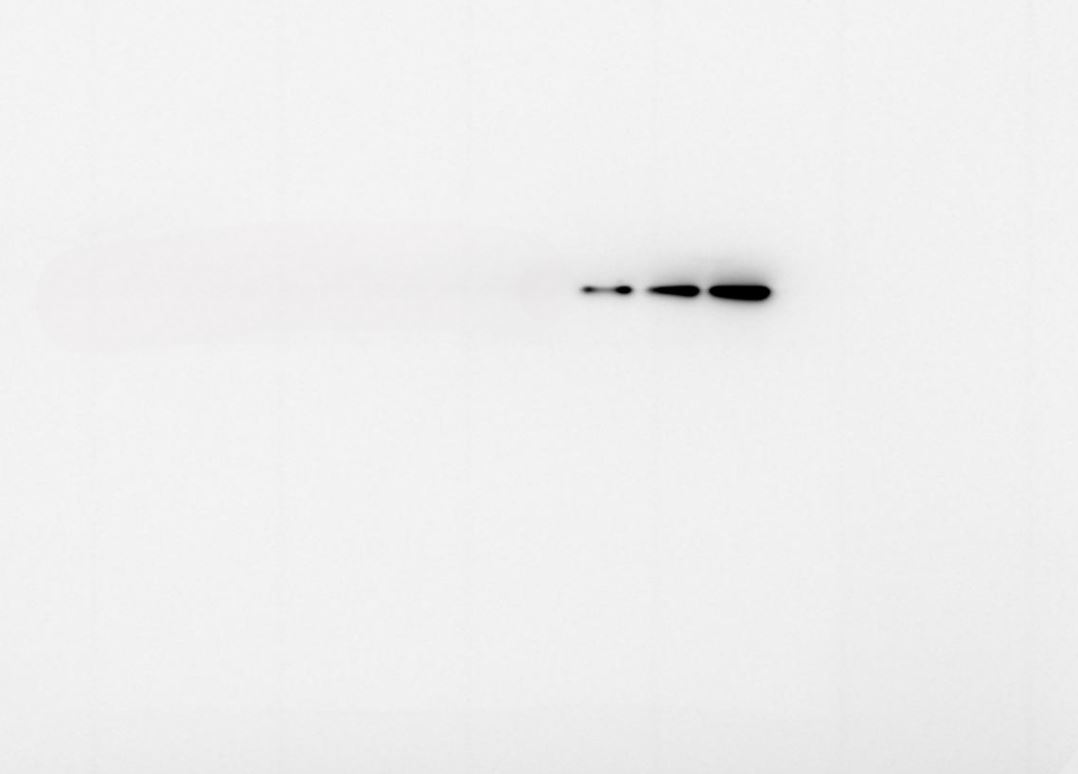


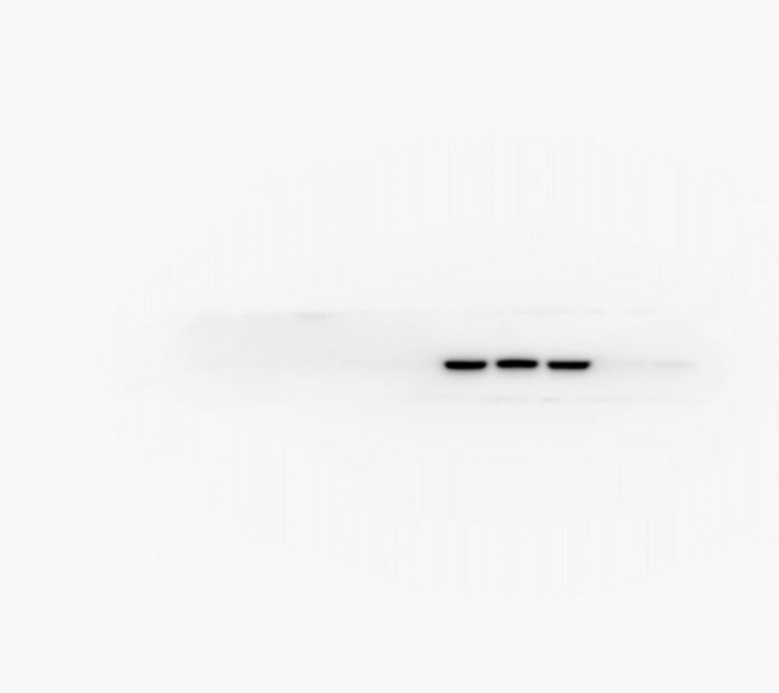


**Figure5**

Figure 5J

Row 6-8


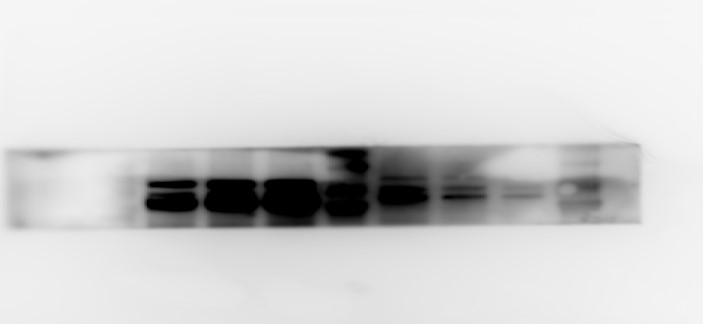


Left panel


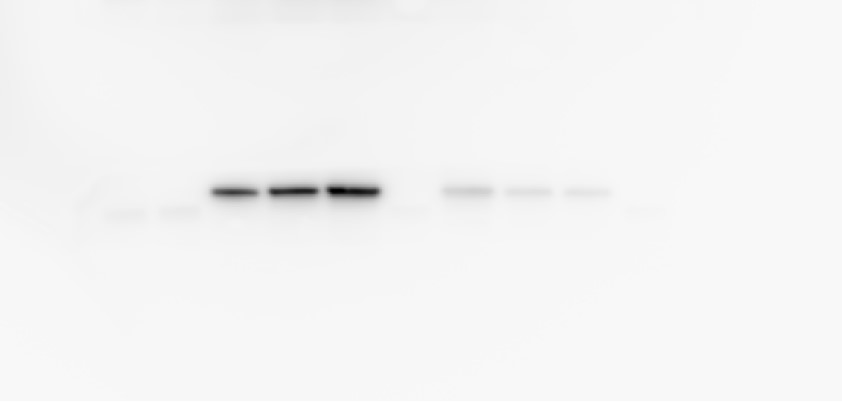


Left panel


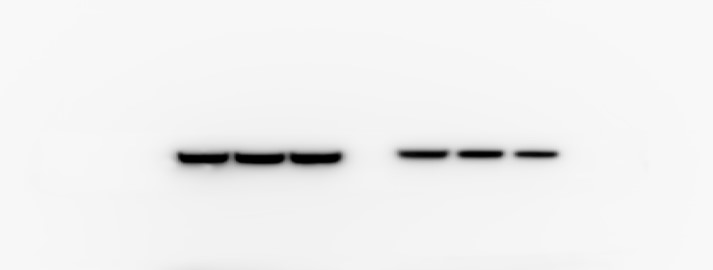


**Figure6**

Figure 6A


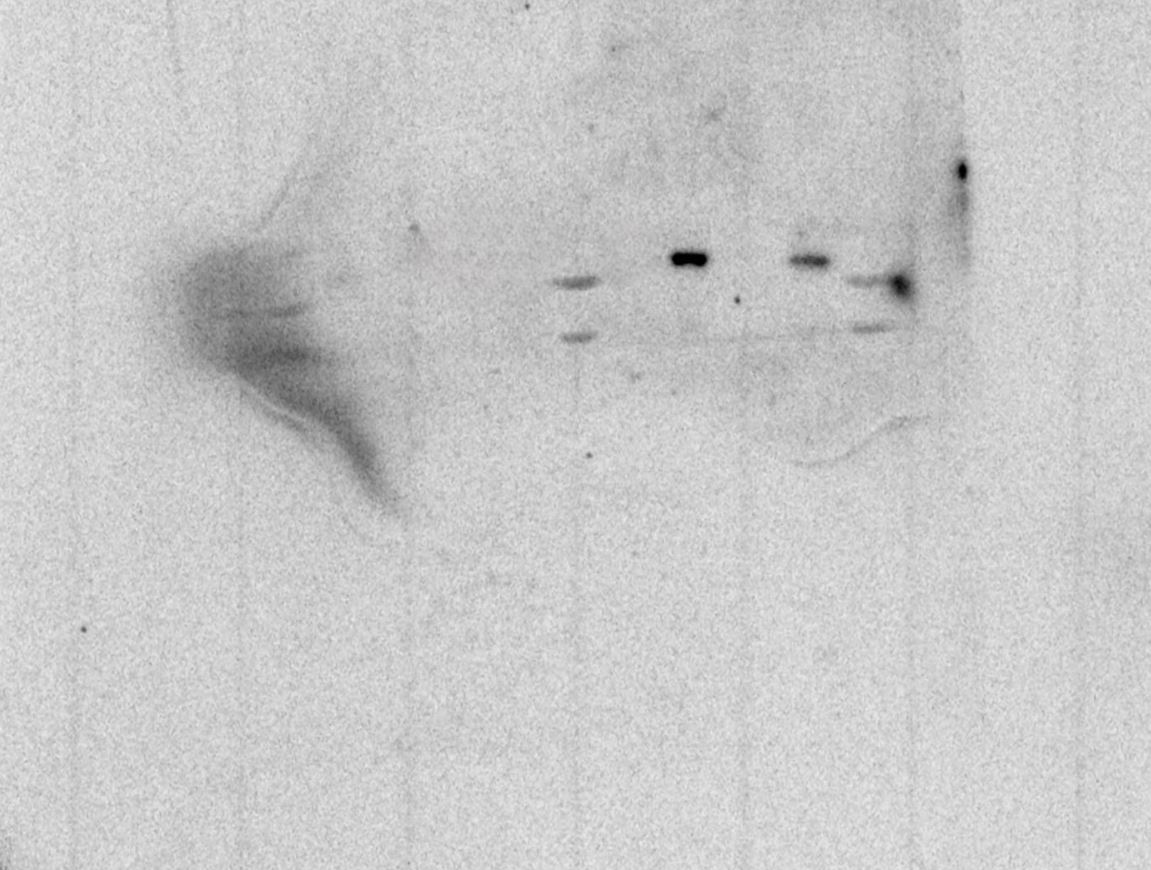


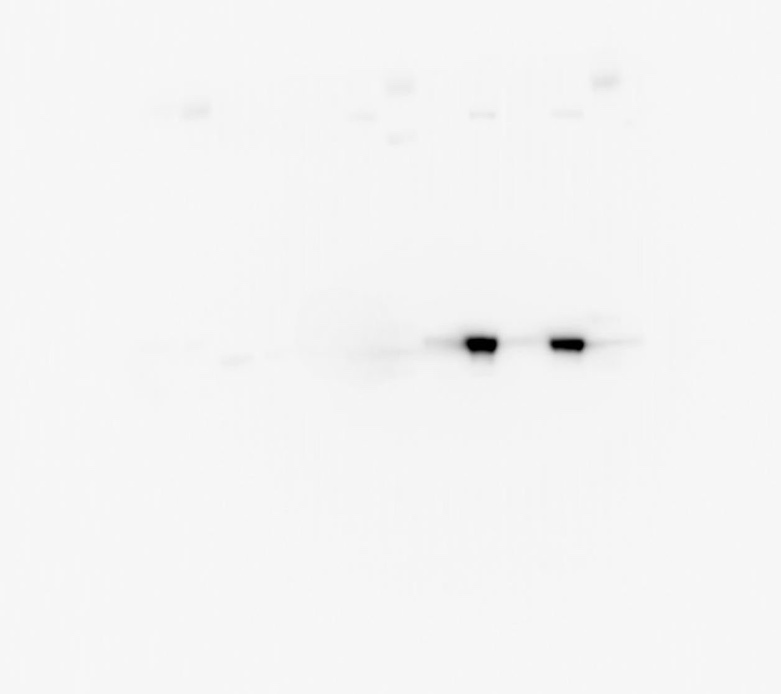


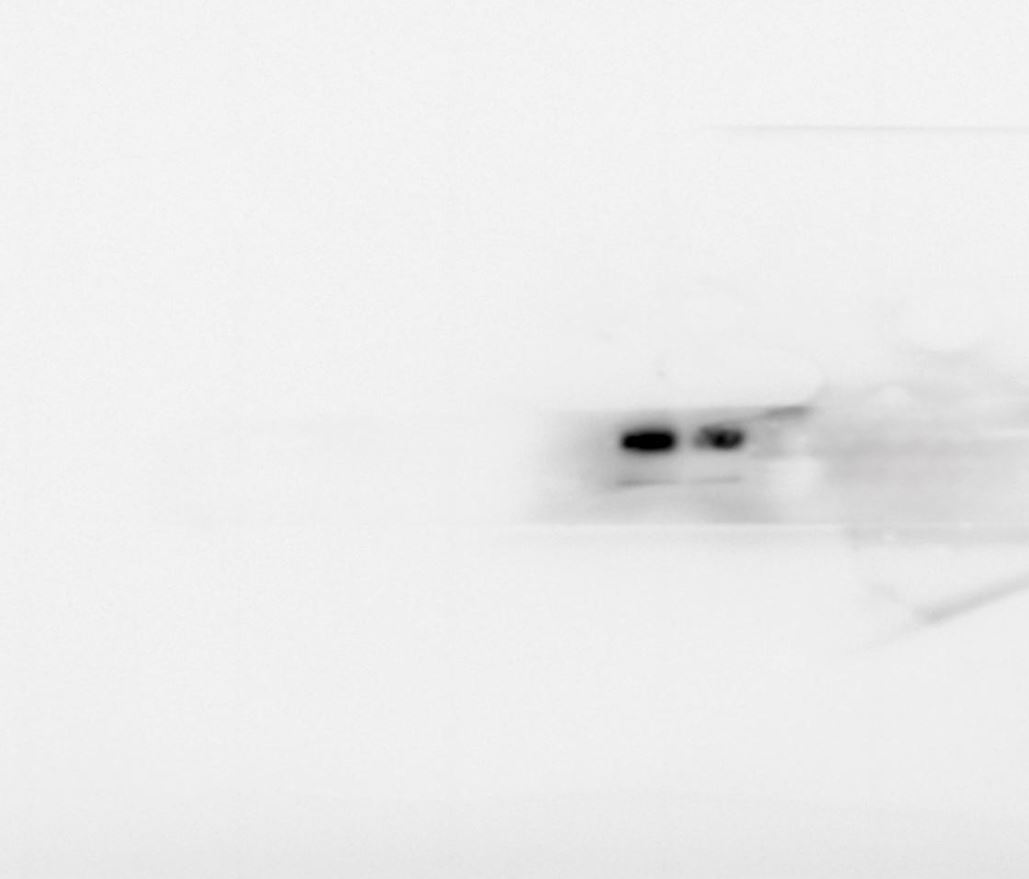


Figure 6B


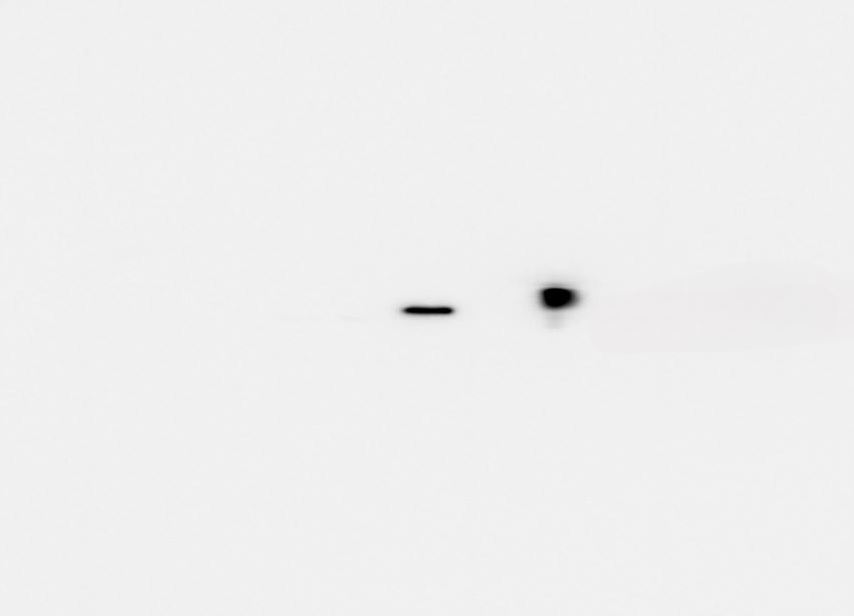


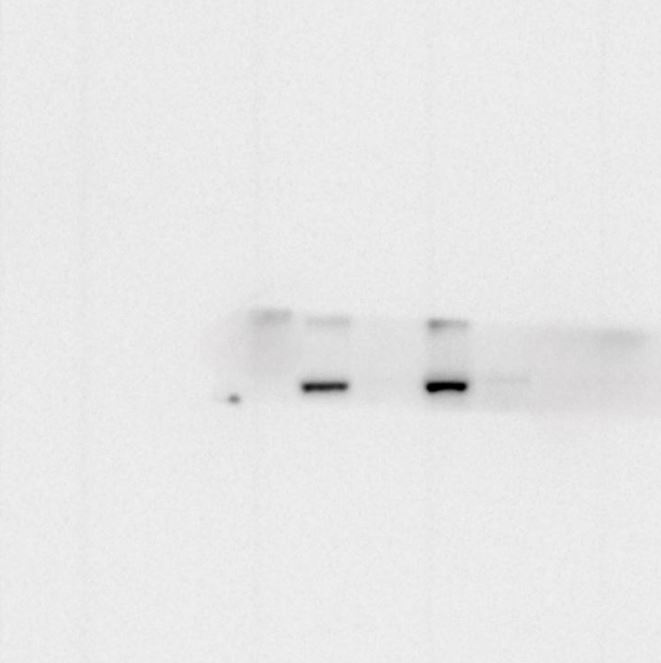


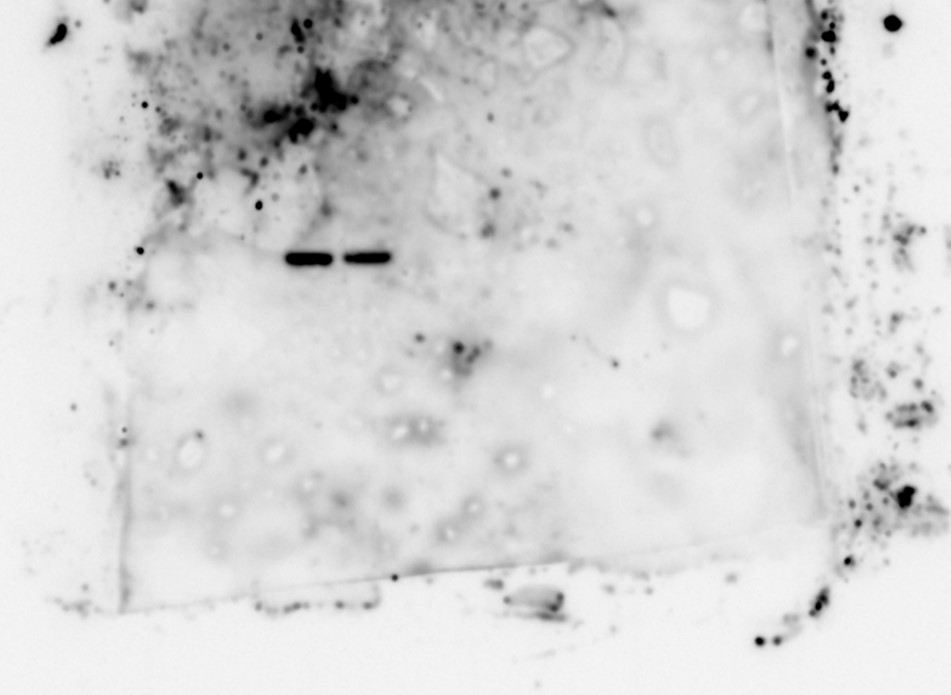


Figure 6C


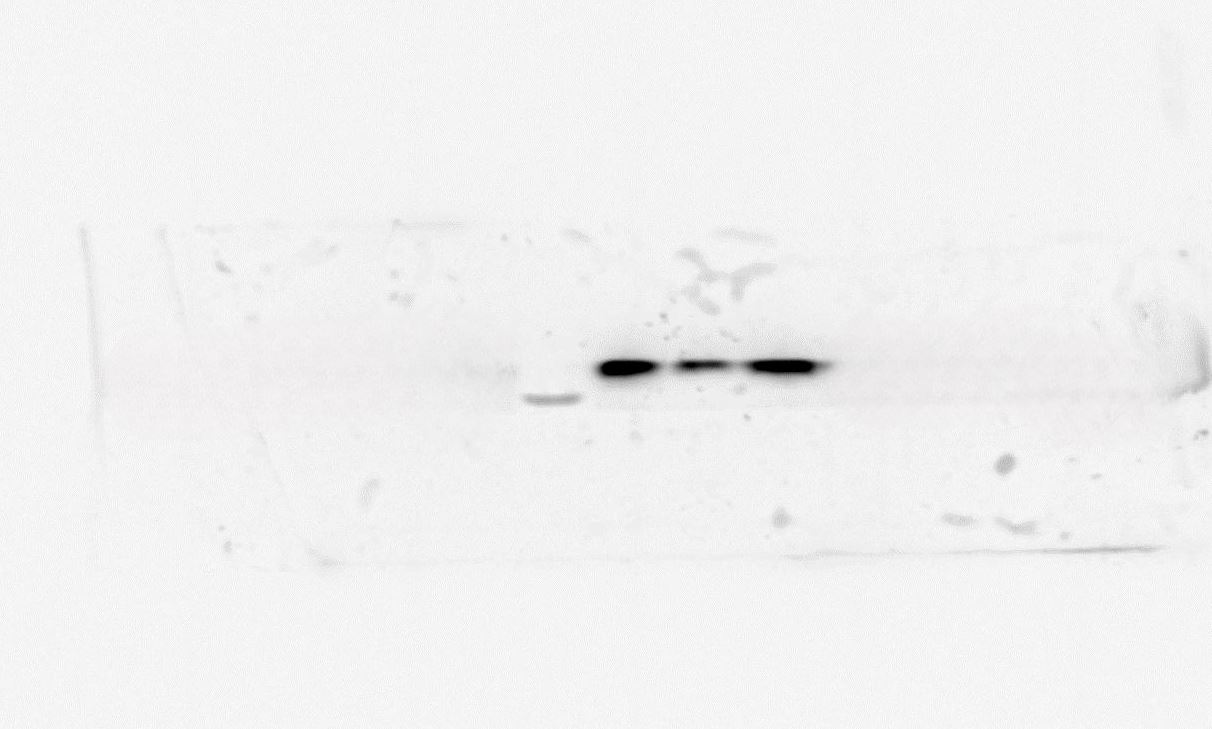


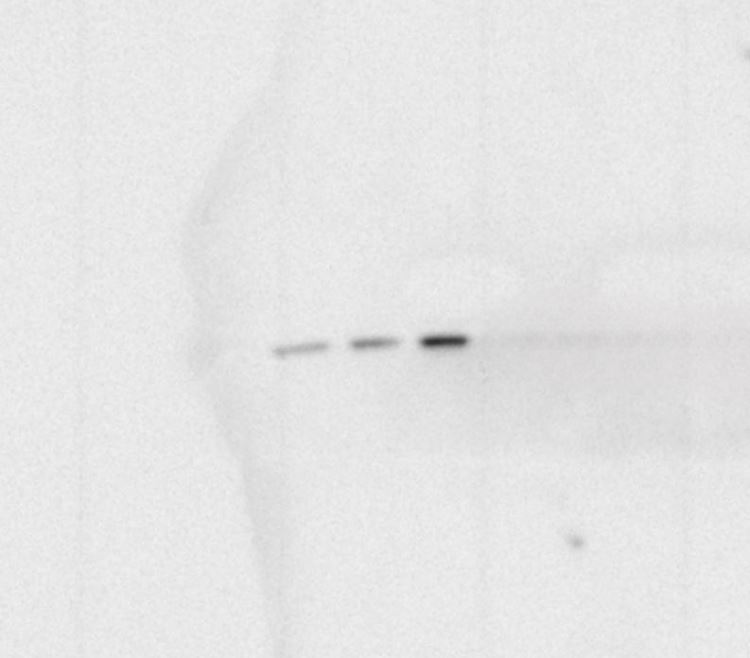


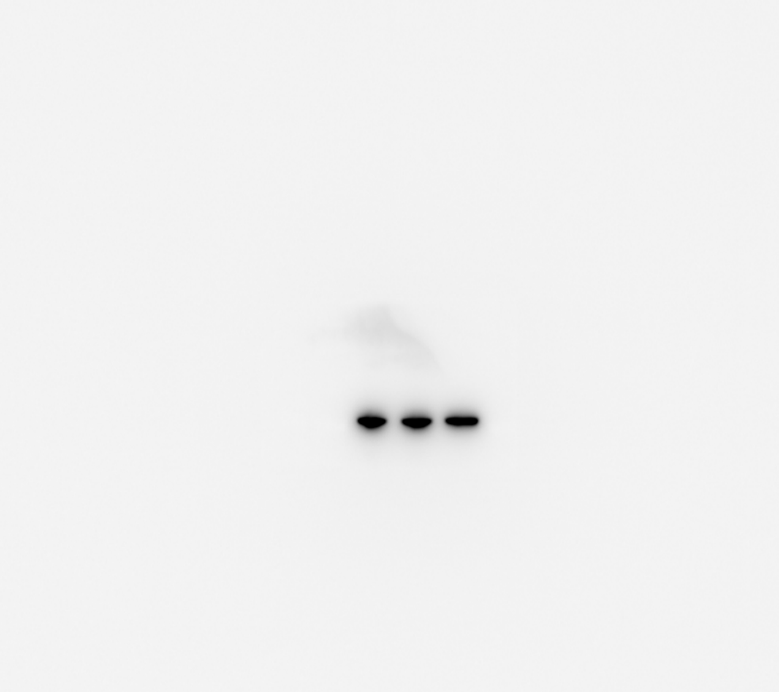


Figure 6D


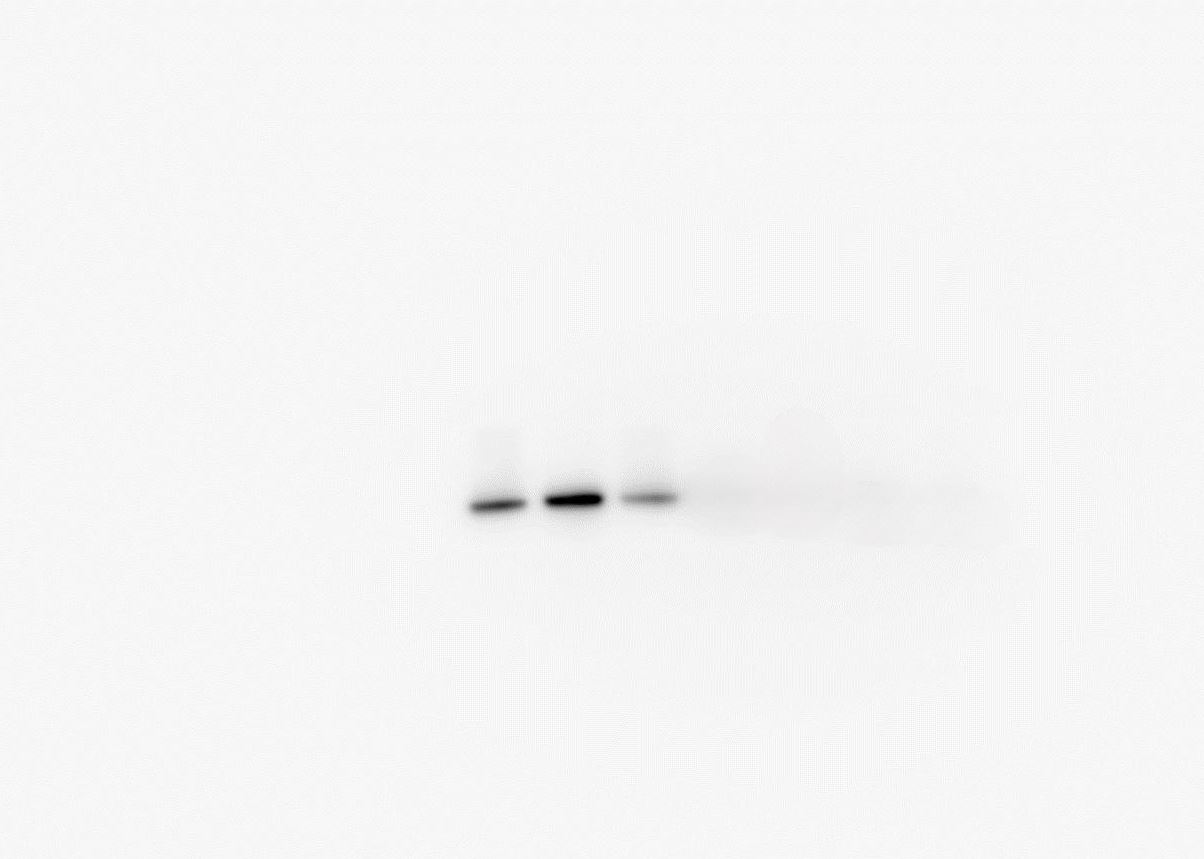


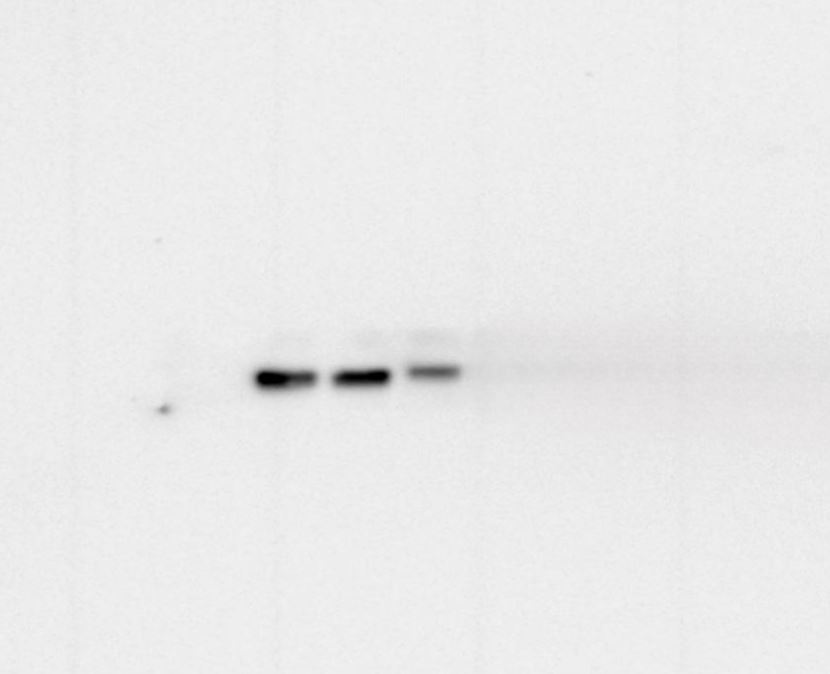


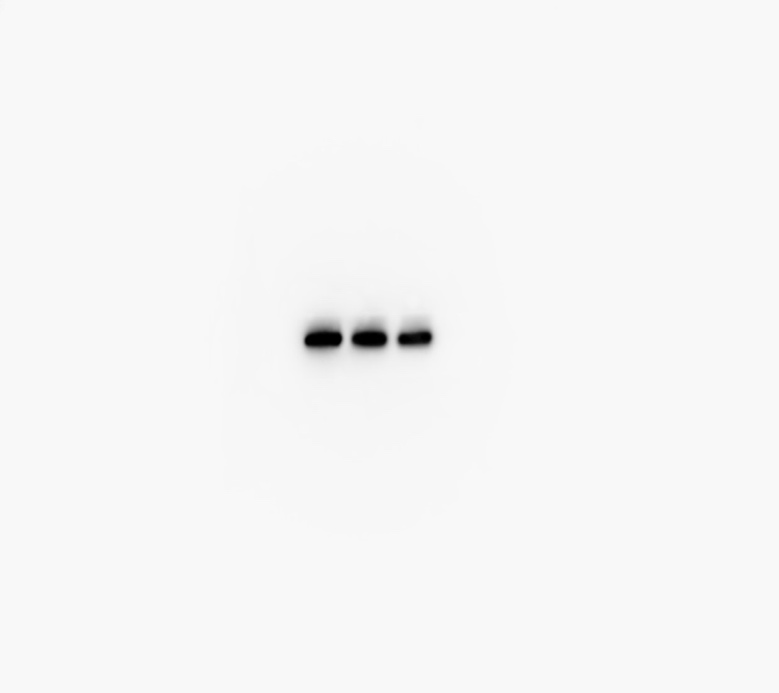


Figure 6E left panel


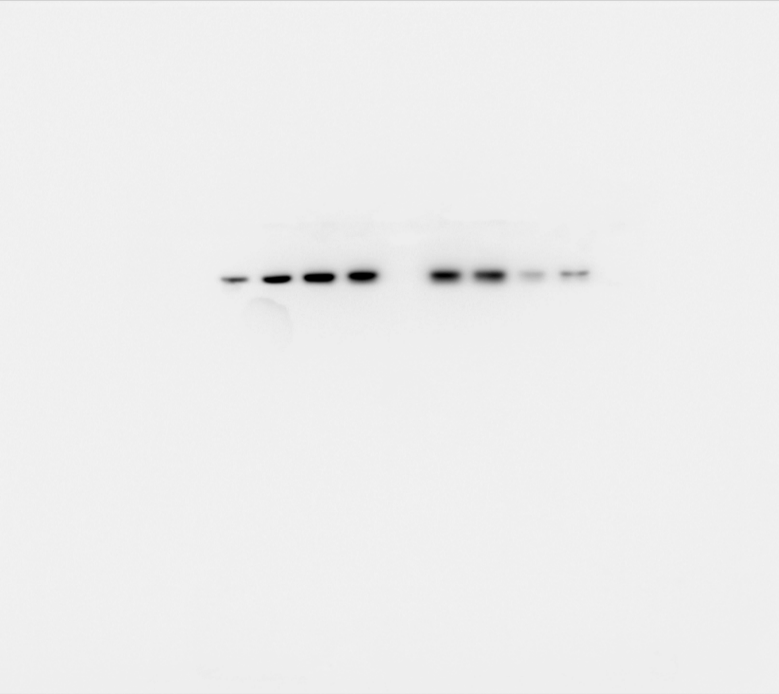


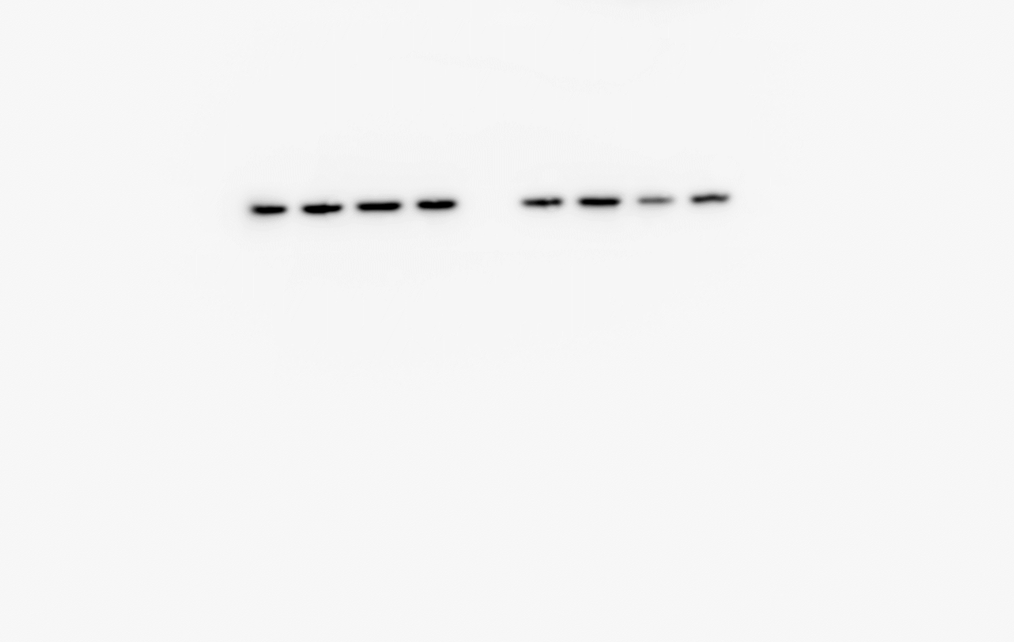


Figure 6F


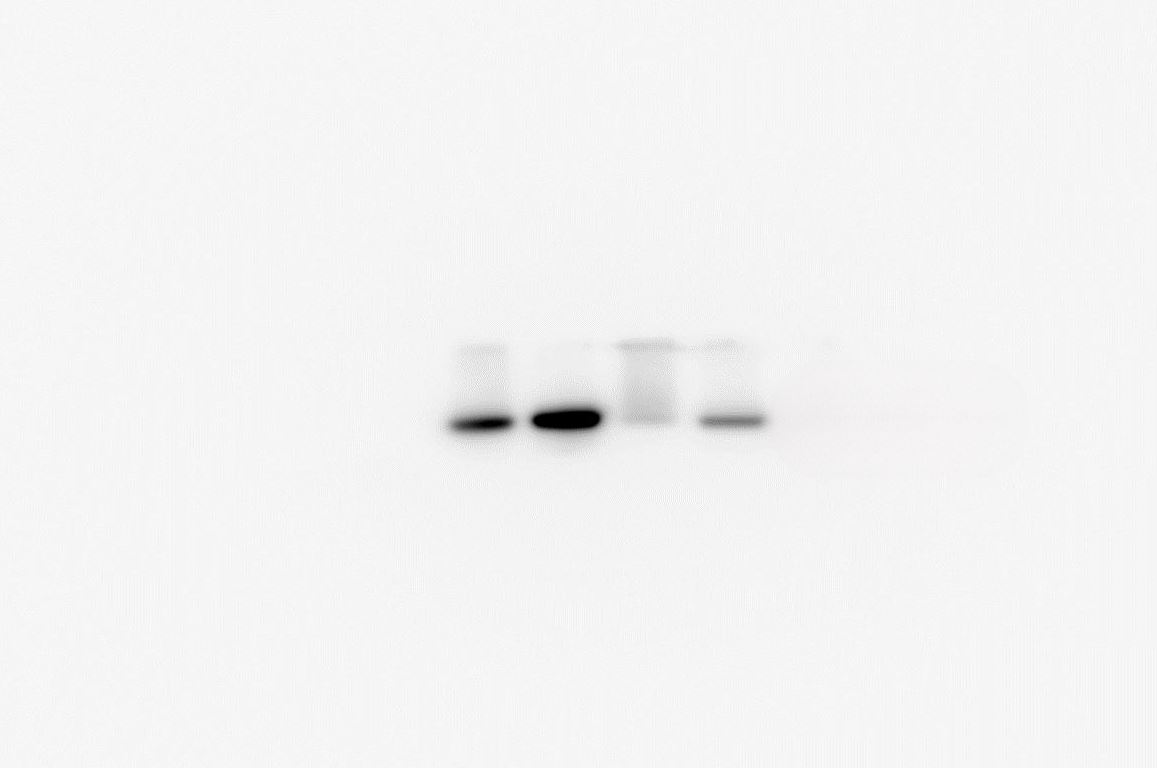


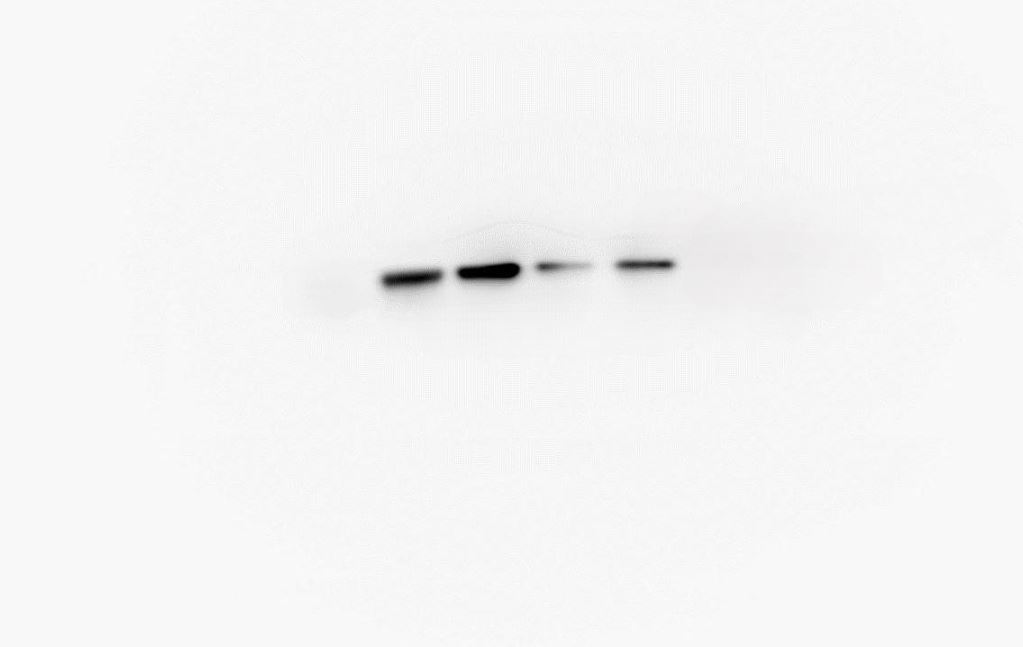


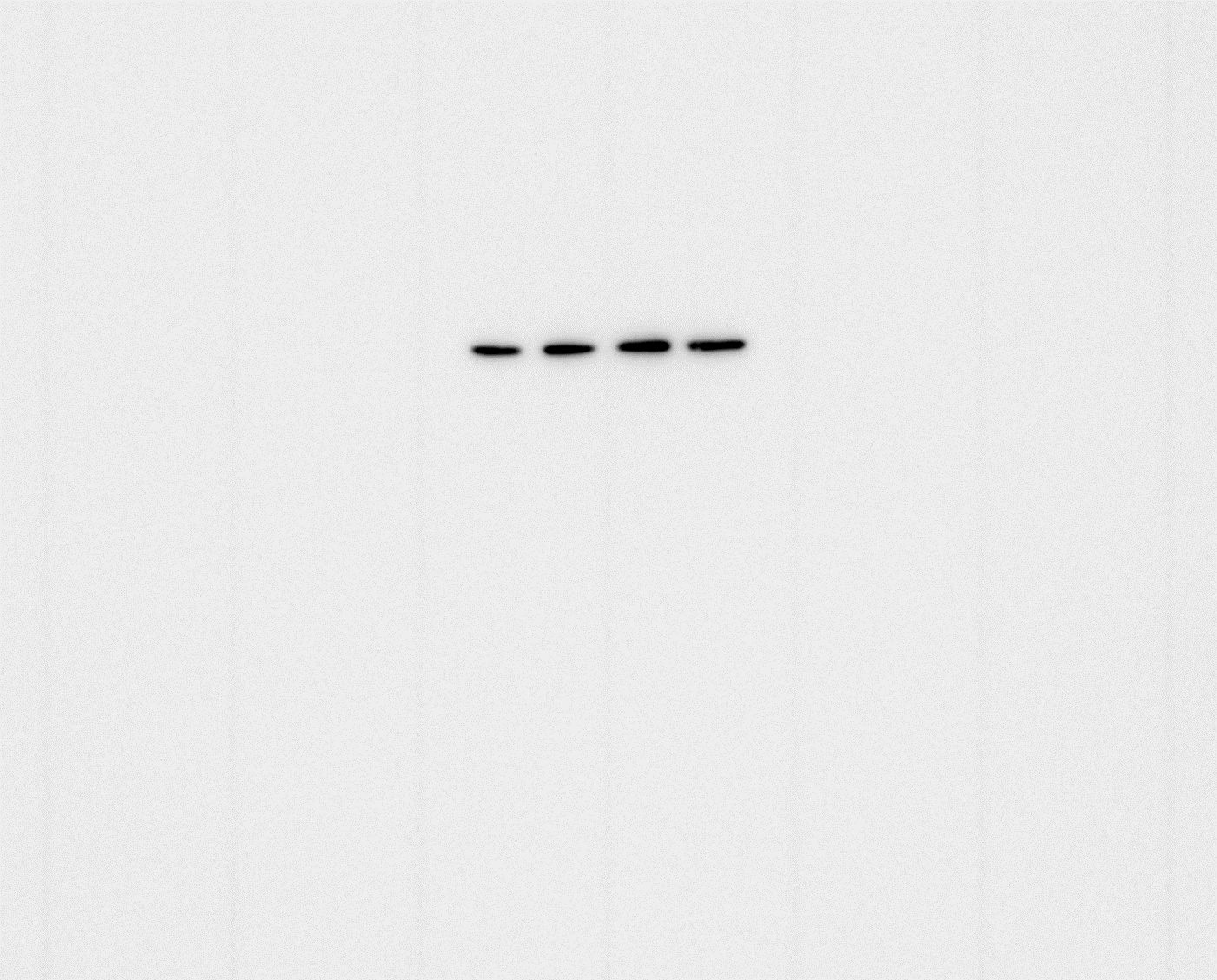


Figure 6G


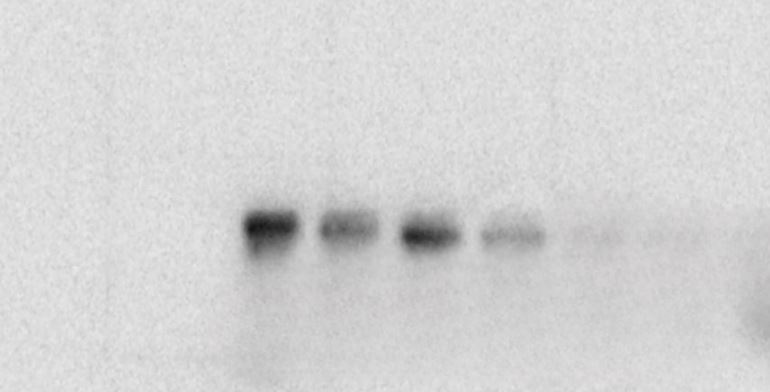


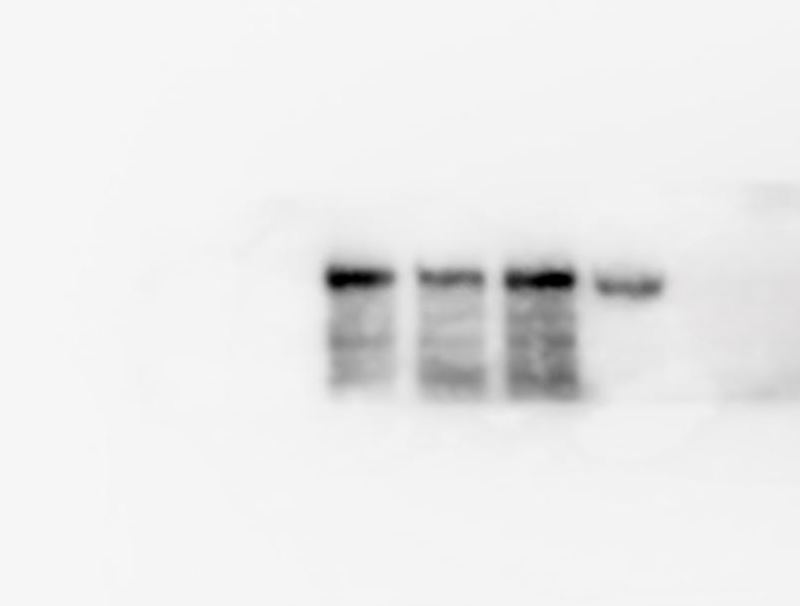


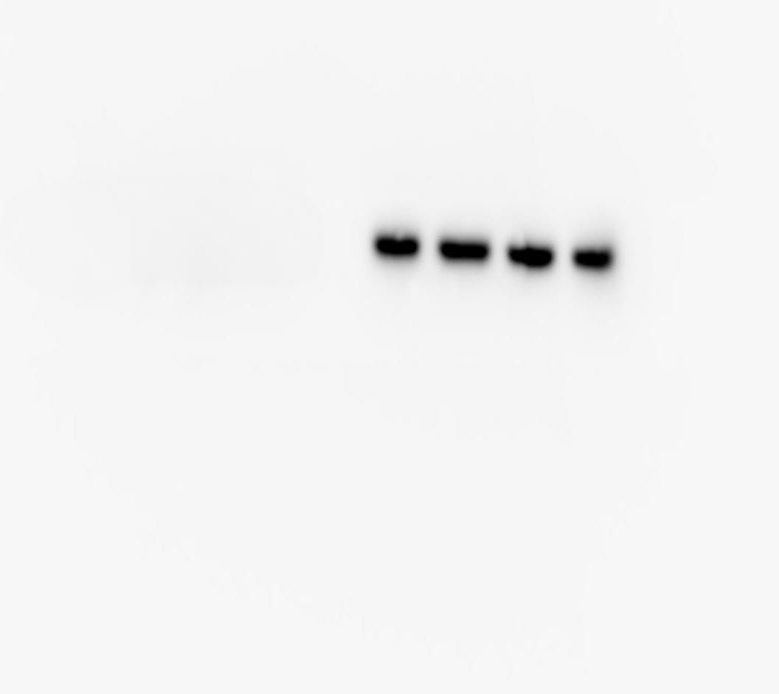


Figure 6H


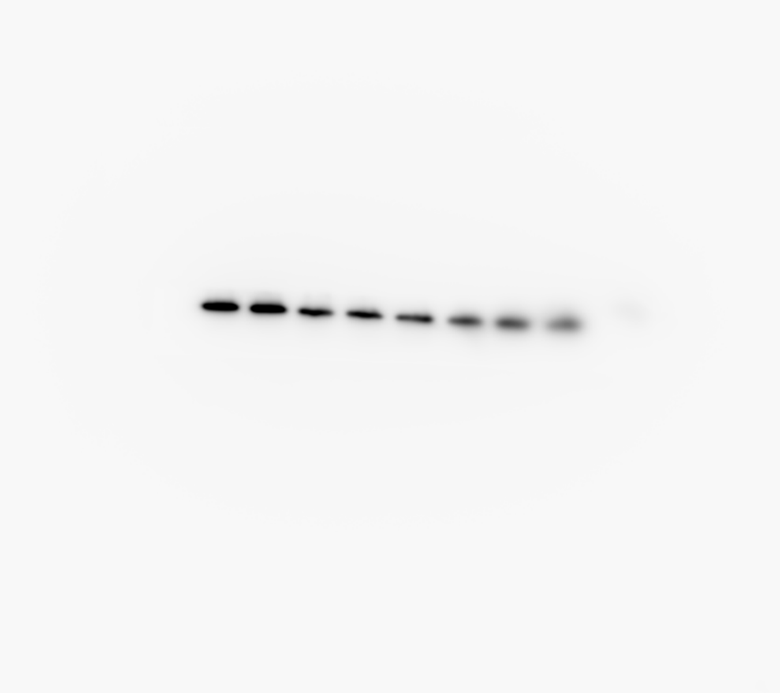


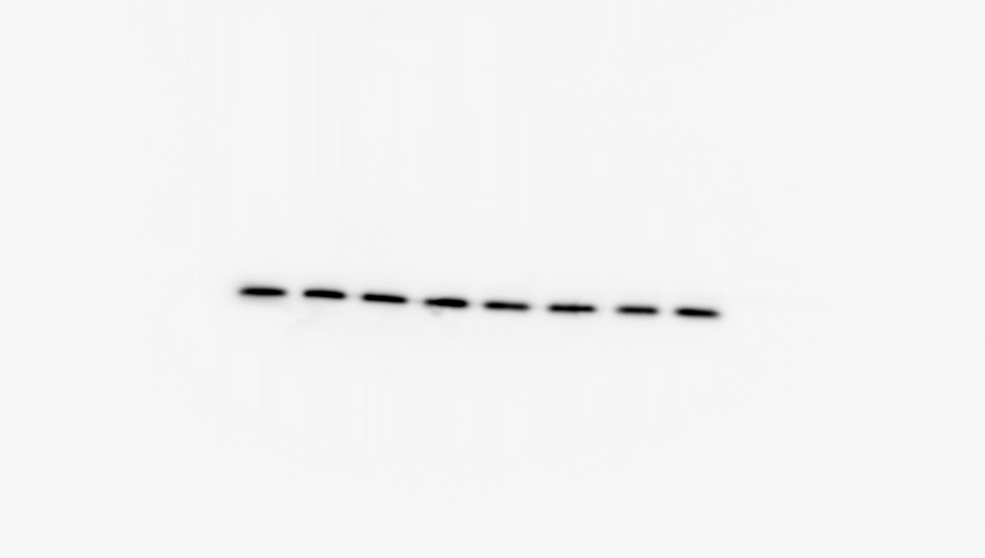

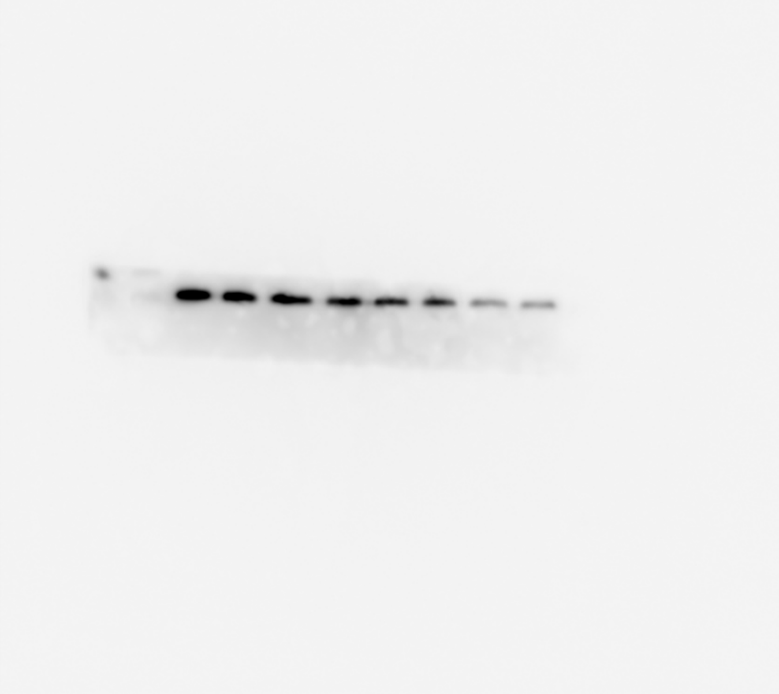


Figure 6I left panel


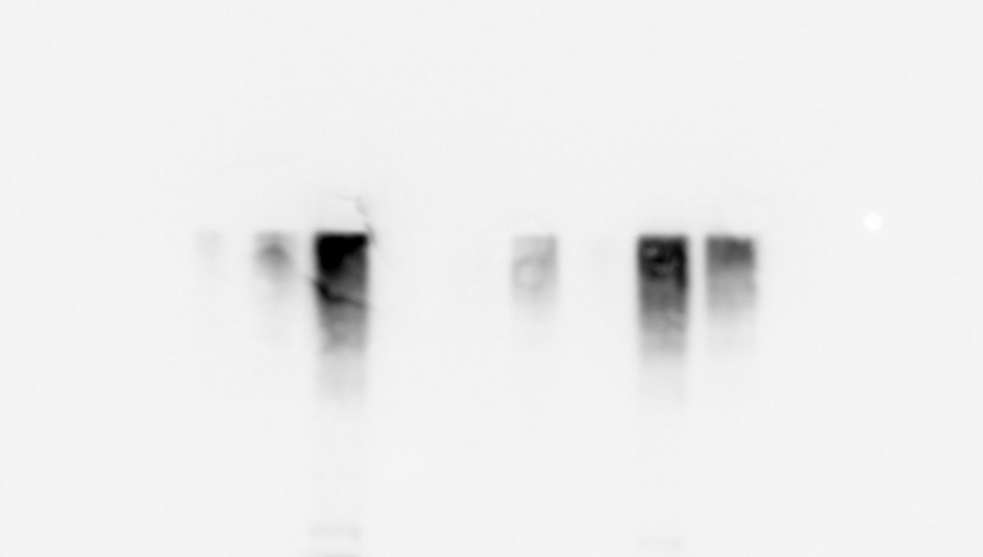


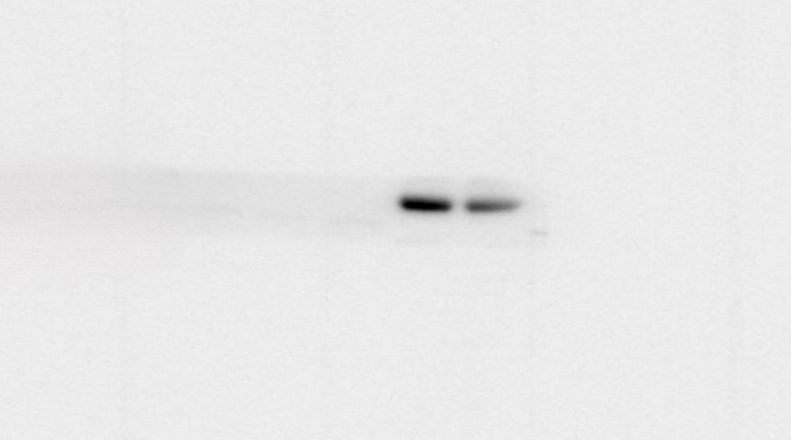


Left panel


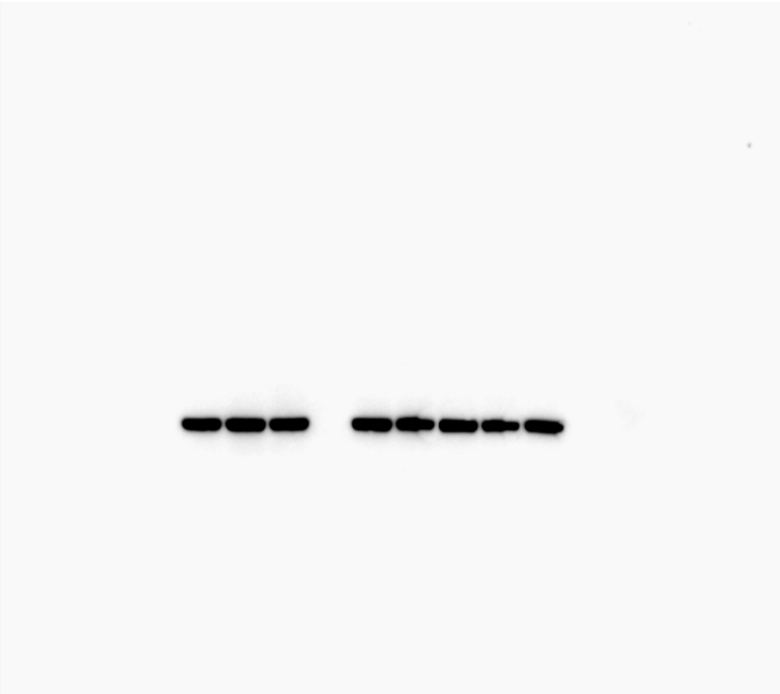


Figure 6J Right panel


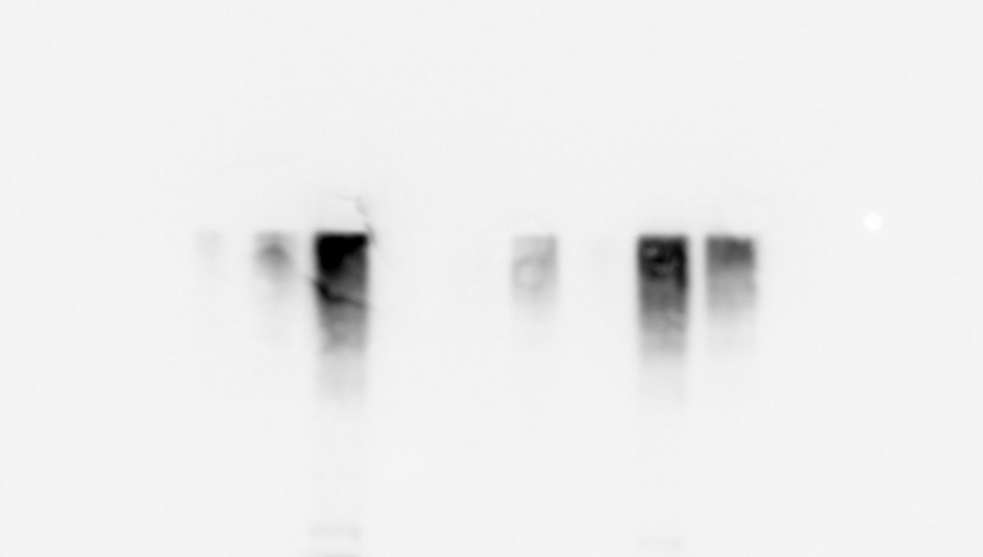


Right panel


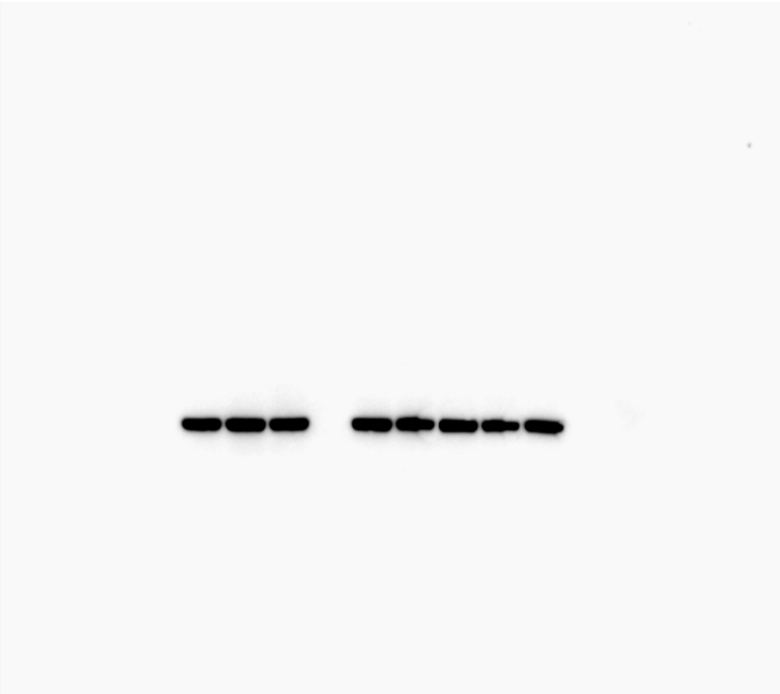


Figure 6K


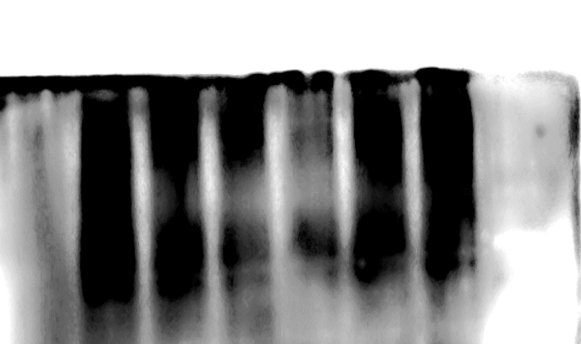


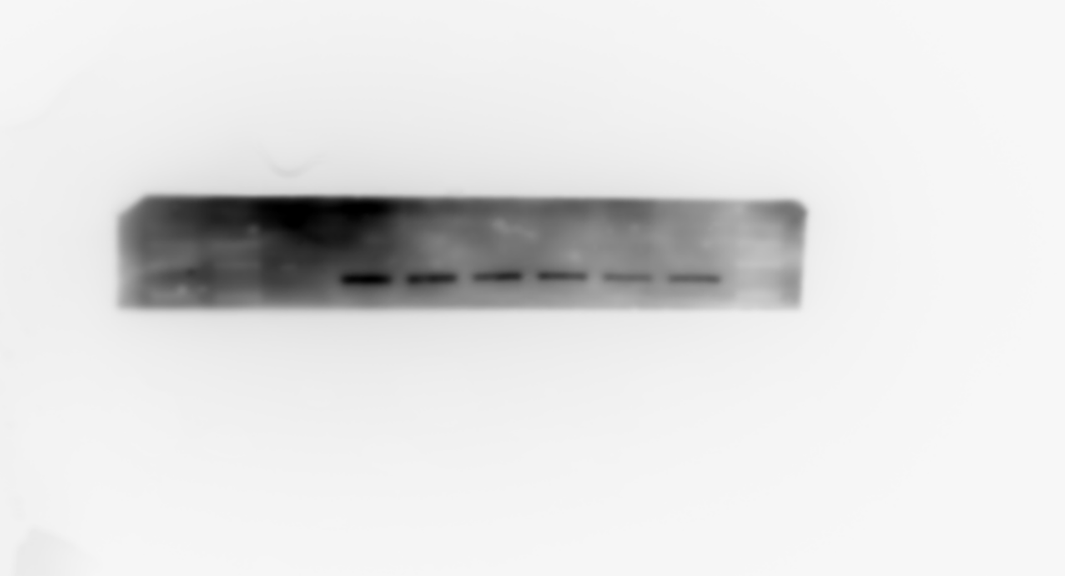


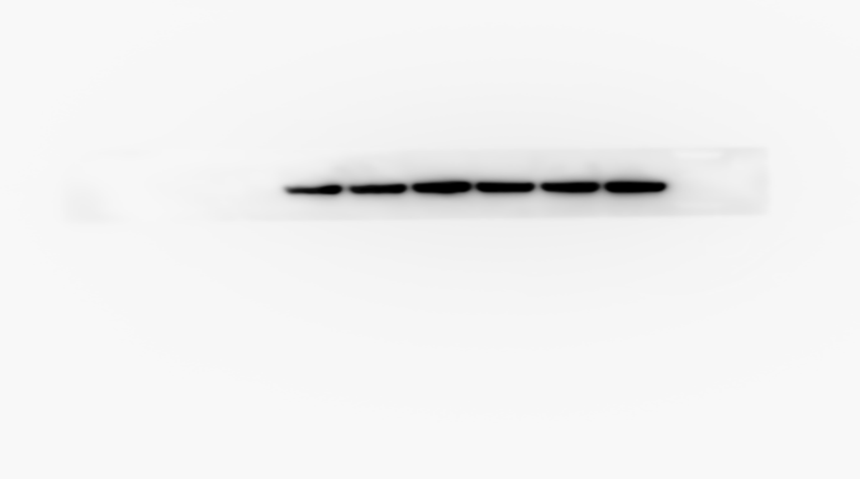


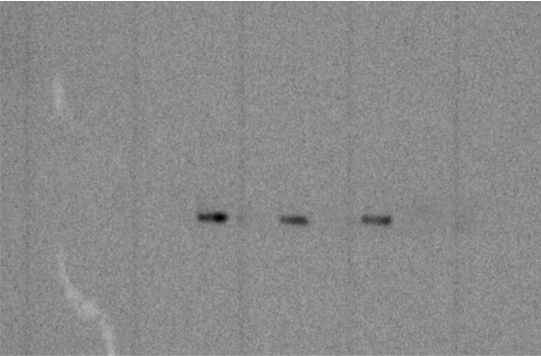


Figure 6L Middle panel


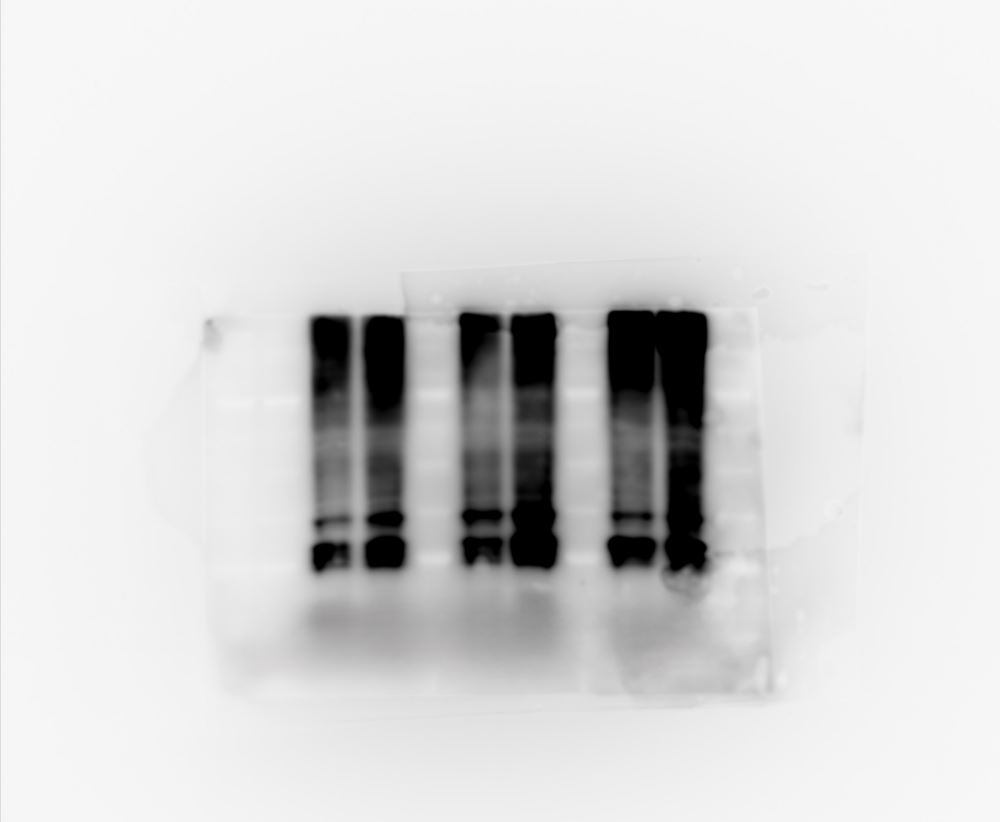


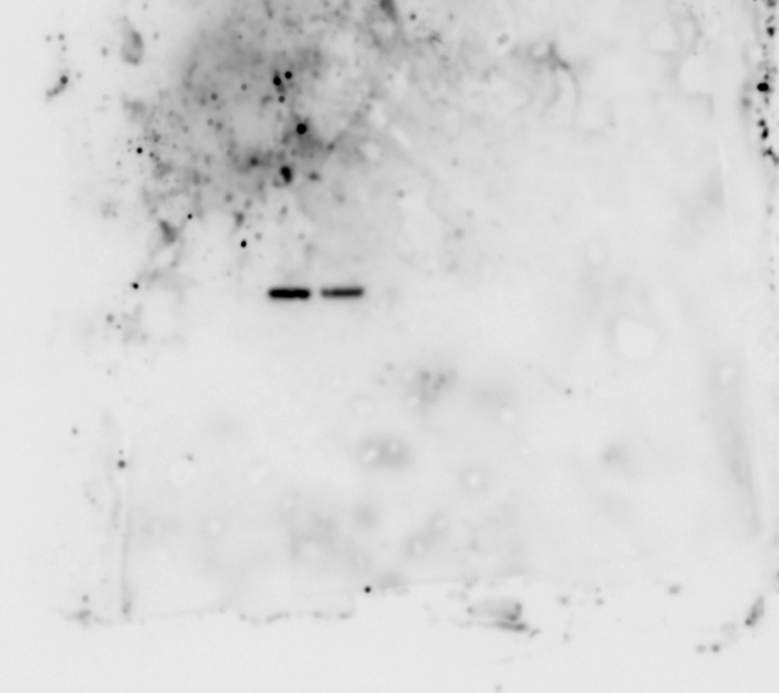


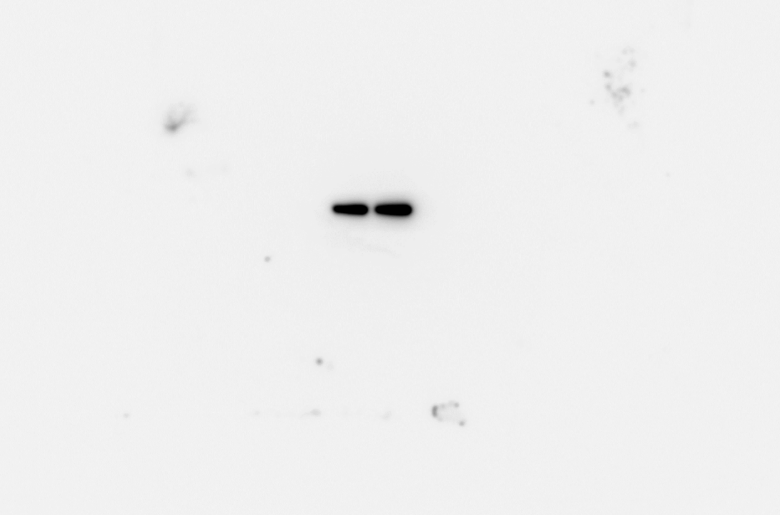


Figure 6M


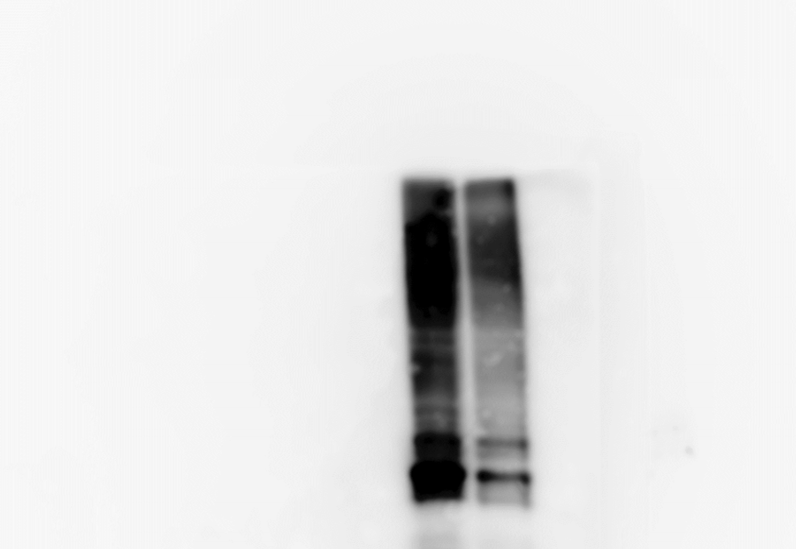


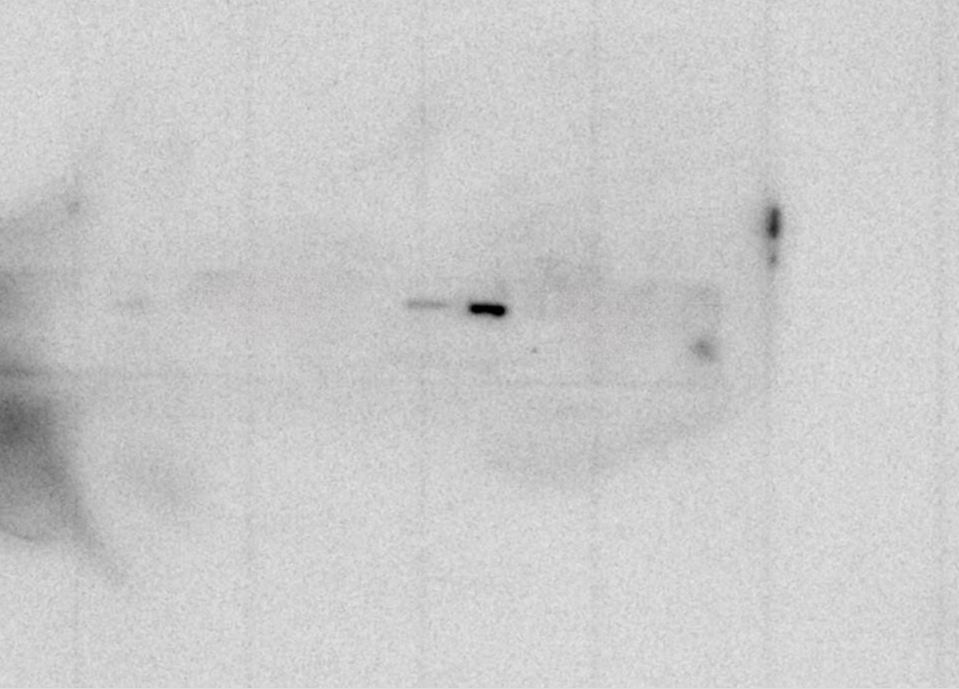


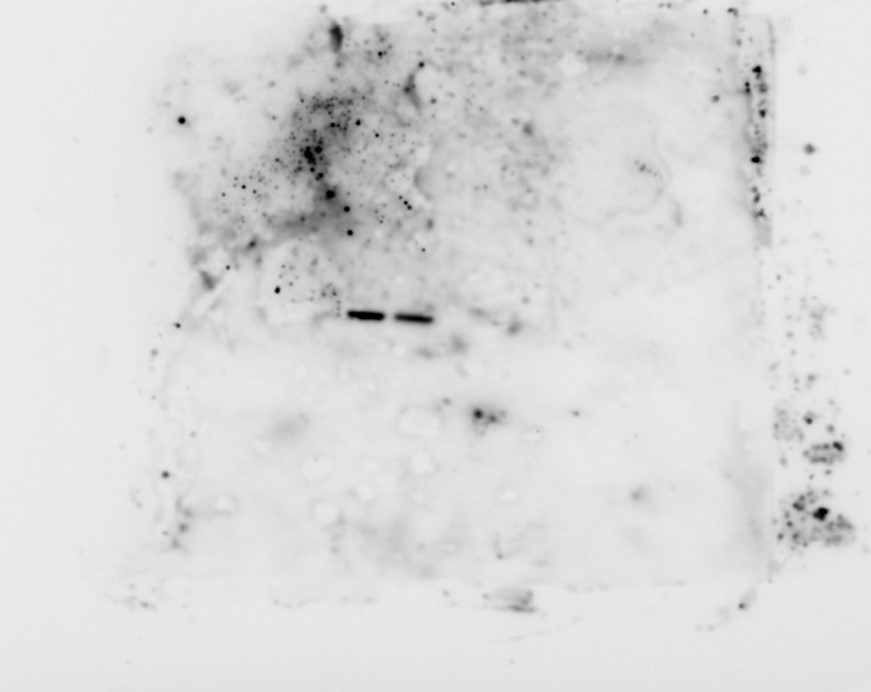

Supplement: Supplementary file 3 — WB [file 41420_2024_1948_MOESM3_ESM.docx]
